# Prostasin regulates PD-L1 expression in human lung cancer cells

**DOI:** 10.1042/BSR20211370

**Published:** 2021-07-09

**Authors:** Li-Mei Chen, Julius C. Chai, Bin Liu, Tara M. Strutt, K. Kai McKinstry, Karl X. Chai

**Affiliations:** 1 Division of Cancer Research, Burnett School of Biomedical Sciences, University of Central Florida College of Medicine, Orlando, FL, U.S.A.; 2 Department of Epigenetics and Molecular Carcinogenesis, Division of Basic Science Research, The University of Texas MD Anderson Cancer Center, Smithville, TX, U.S.A.; 3 Division of Immunity and Pathogenesis, Burnett School of Biomedical Sciences, University of Central Florida College of Medicine, Orlando, FL, U.S.A.

**Keywords:** CD274, extracellular vesicles, immune checkpoint, interferon-gamma, PRSS8

## Abstract

The serine protease prostasin is a negative regulator of lipopolysaccharide-induced inflammation and has a role in the regulation of cellular immunity. Prostasin expression in cancer cells inhibits migration and metastasis, and reduces epithelial–mesenchymal transition. Programmed death-ligand 1 (PD-L1) is a negative regulator of the immune response and its expression in cancer cells interferes with immune surveillance. The aim of the present study was to investigate if prostasin regulates PD-L1 expression. We established sublines overexpressing various forms of prostasin as well as a subline deficient for the prostasin gene from the Calu-3 human lung cancer cells. We report here that PD-L1 expression induced by interferon-γ (IFNγ) is further enhanced in cells overexpressing the wildtype membrane-anchored prostasin. The PD-L1 protein was localized on the cell surface and released into the culture medium in extracellular vesicles (EVs) with the protease-active prostasin. The epidermal growth factor-epidermal growth factor receptor (EGF-EGFR), protein kinase C (PKC), and mitogen-activated protein kinase (MAPK) participated in the prostasin-mediated up-regulation of PD-L1 expression. A Gene Set Enrichment Analysis (GSEA) of patient lung tumors in The Cancer Genome Atlas (TCGA) database revealed that prostasin and PD-L1 regulate common signaling pathways during tumorigenesis and tumor progression.

## Introduction

Lung cancer remains the second most diagnosed cancer for both men and women, and the leading cause of cancer-related deaths in the United States, with 235760 new cases and 131880 deaths estimated for 2021 [1]. These current facts and figures represent an annual decrease of 2% in incidents since the mid-2000s, and a greater decline in percent deaths since 1990, as a result of smoking cessation campaigns and improved diagnosis and treatments. Non-small cell lung cancers (NSCLCs) constitute the majority of lung cancers, at 84%. Surgery, chemotherapy and radiation are the treatment options for early-stage NSCLCs. Advanced-stage patients are treated with chemotherapy, molecular-targeting drugs and/or immunotherapy. Despite the efforts and advances, the 5-year survival rate for lung cancer remains low at 21% overall and 25% for NSCLCs. Localized lung cancers have a 59% 5-year survival rate but only 17% of lung cancers are diagnosed at this stage. New drug targets and treatment strategies are required to further improve lung cancer survival.

Immunotherapy regimes targeting the immune checkpoint molecules programmed cell death protein 1 (PD-1) and programmed death-ligand 1 (PD-L1, CD274) are showing great promises in treating lung cancers, especially in patients with pretreated advanced NSCLC. The main functions of PD-L1 are the inhibition of T-cell proliferation, induction of immune cell apoptosis, and suppression of T-cell cytokine secretion. These actions are mediated by PD-L1 binding to its receptor PD-1, which is expressed on activated T cells, B cells and myeloid cells. This mechanism normally acts in tissues to limit autoimmune reactions or immune destruction that could be caused by overly robust inflammatory responses [2–4]. However, this pathway is also used by tumor cells to evade immune elimination and can promote tumor progression [5,6]. The cytokine interferon-γ (IFNγ) is a strong inducer of PD-L1 expression [7]. Tumor-infiltrating lymphocytes present in the tumor microenvironment are a major source of IFNγ to increase tumor cell PD-L1 expression [8,9].

Nivolumab (targeting PD-1), pembrolizumab (targeting PD-1) and atezolizumab (targeting PD-L1) are examples of blocking antibodies to overcome this regulatory blockade during cancer treatment, in combination with other regimens. Clinical trials in pretreated advanced NSCLC patients indicated a longer median overall survival (OS), a higher median duration of response (DOR), a longer median progression-free survival (PFS), and a higher median overall response rate (ORR) when compared with the standard-of-care chemotherapy, e.g., docetaxel [10].

The trypsin-like serine protease prostasin (PRSS8) is extracellularly tethered on the epithelial cell membrane via a glycosylphosphatidylinositol (GPI) anchor [11–13]. Prostasin is expressed in all normal epithelial cells and low-grade tumors, but often down-regulated in high-grade tumors [14] and during inflammation [15]. We have previously established a role for prostasin in reducing inflammation [15–17], suppressing tumor cell invasion and metastasis [18–20], and in inhibiting the epithelial–mesenchymal transition [21]. We have also identified prostasin as a regulator of cytokine and reactive oxygen species production including IFNγ, tumor necrosis factor α (TNF-α) and the inducible nitric oxide synthase (iNOS), all of which are implicated for a role in the tumor microenvironment and progression [22].

In the present study, we intended to investigate if prostasin, as a regulator of the inflammatory cytokines, has a role in the regulation of PD-L1 expression. A human NSCLC cell line, Calu-3, was used as a model to establish stable sublines expressing the human prostasin or its functional variants via lentiviral transduction. A stable subline with the prostasin gene knocked-out via gene editing was also established. In these stable Calu-3 sublines, we evaluated PD-L1 expression changes with or without IFNγ stimulation. The signaling pathways mediating prostasin regulation of PD-L1 expression were investigated, including the epidermal growth factor (EGF)-epidermal growth factor receptor (EGFR) axis, protein kinase C (PKC), and the mitogen-activated protein kinase (MAPK). The relationship of prostasin and PD-L1 in lung tumors was further explored in patient specimens using data from The Cancer Genome Atlas (TCGA).

## Materials and methods

### Cell culture

The Calu-3 (ATCC® HTB-55™) human lung adenocarcinoma cell line, the Beas-2B (ATCC® CRL-9609™) human normal lung epithelial cell line, and the PC-3 (ATCC® CRL-1435™) and DU-145 (ATCC® HTB-81™) human prostate carcinoma cell lines were purchased from the American Type Culture Collection (ATCC, Manassas, VA). The human telomerase reverse transcriptase (hTERT)-immortalized B6Tert-1 normal human trophoblast cell line was a gift from Dr. Yanling Wang (Institute of Zoology, Beijing, China) [23]. Tissue culture flasks and dishes were purchased from Sarstedt, Inc. (Newton, NC). Transwell® inserts for air–liquid cell cultures were purchased from Corning Inc. (Corning, NY). Fetal bovine serum (FBS) was purchased from Sigma–Aldrich (St. Louis, MO). Other cell culture media and reagents were purchased from Thermo Fisher Scientific (Waltham, MA). The parent Calu-3 cells and the genetically engineered sublines were cultured in Eagle’s minimum essential medium (EMEM) supplemented with 10% FBS and sodium pyruvate. The Beas-2B cells were cultured in the bronchial epithelial cell growth basal medium (BEBM, Cat. No. CC-3171, Lonza/Clonetics Corporation, Basel, Switzerland) supplemented with the additives from the BEGM Kit (Cat. No. CC-3130, Lonza/Clonetics). The PC-3 and DU-145 cells were maintained as described previously [18]. All cells were incubated at 37°C in a humidified atmosphere of 5% CO_2_ in air.

### Chemicals and antibodies

The PKC inhibitor Gö 6976 (selective for PKCα and PKCβ1, Product No. 365253-1ML) and MAPK/ERK kinase (MEK) inhibitor U0126 (Product No. 19-147) were purchased from Calbiochem/MilliporeSigma (St. Louis, MO). The tyrosine kinase inhibitor (TKI) lapatinib was purchased from Santa Cruz Biotechnology (Dallas, TX, Cat. No. sc-202205).

The commercial antibodies were purchased from Cell Signaling Technology, Inc. (Danvers, MA): PD-L1 (Cat. No. 13684P), pSTAT1 (Cat. No. 8826S, 7649S), total STAT1 (Cat. No. 9175S), pERK1/2 (Cat. No. 9101S); or Santa Cruz Biotechnology: GAPDH (Cat. No. sc-32233), EGFR (Cat. No. sc-03), total ERK (Cat. No. sc-94), CD63 (Cat. No. sc-5275), Alix (Cat. No. sc-53540), Tsg101 (Cat. No. sc-7964), HSP70 (Cat. No. sc-24); or MilliporeSigma (St. Louis, MO): CMTM6 (Cat. No. HPA026980), β-tubulin (Cat. No. T4026); pPKCα (Cat. No. 06-822); or BD Biosciences (San Jose, CA): PKCα (Cat. No. 610107). The human prostasin antibody was described previously [13]. The anti-EGFR monoclonal antibody cetuximab/Erbitux was generously provided by ImClone Systems (Bridgewater, New Jersey) and the anti-Her2 monoclonal antibody trastuzumab/Herceptin was generously provided by Genentech (South San Francisco, CA). For all Western blots the antibodies were used at the dilution ratio of 1:1000.

Antibodies used for flow cytometry: Allophycocyanin (APC)-conjugated mouse anti-human CD274 (B7-H1 PD-L1) antibody (BioLegend, San Diego, CA; #329708, clone 29E.2A3, mouse IgG2b, *k*), APC mouse IgG2b, *k* isotype control antibody (BioLegend, #400322, clone MPC-11), goat anti-rabbit IgG-cyanine Cy™2 fluorophore-conjugated secondary antibody (Jackson ImmunoResearch Laboratories, Inc., West Grove, PA; Code: 111-225-144).

### Establishment of Calu-3 sublines

The cDNAs coding for the wildtype human prostasin and the protease-dead variant were described previously [23]. The prostasin cDNA in the lentiviral vector contained only the coding sequence (nucleotides 230–1261 in NM_002773). The cDNA coding for the GPI-anchor-free prostasin variant was engineered via PCR-mediated deletion of the GPI-anchor signal coding sequence (nucleotides 1196–1258). The cDNAs coding for prostasin and the variants were subcloned into the pLVX-Puro vector (Clontech Laboratories, Inc., Mountain View, CA) to produce lentiviruses for transduction of the Calu-3 cells. The CRISPR/Cas9 All-in-One Lentivector set with a PRSS8 gRNA (Cat. No. K1733205) or a Scrambled gRNA (Cat. No. K010) was purchased from Applied Biological Materials Inc. (Richmond, BC, Canada) for knocking out the prostasin gene in the Calu-3 cells. Lentiviral production and transduction of cells were carried out as described previously [23]. Each subline was established by culturing the transduced cells in puromycin (5 µg/ml) for 2 weeks, and maintained as a polyclonal mixture from an initial 50–100 drug-resistant colonies.

### Reverse-transcription and real-time quantitative polymerase chain reaction

Total cellular RNA was extracted using the TRIzol reagent (Invitrogen, Carlsbad, CA) according to the manufacturer’s protocol. One microgram of total RNA from each sample was subjected to reverse-transcription using the iScript cDNA Synthesis Kit (Cat. No. 170-8891, Bio-Rad, Hercules, CA) and one-fifth of the iScript product was used for each gene-specific qPCR, using the iQ SYBR Green Supermix (Cat. No. 170-8882, Bio-Rad). For quantitative comparisons between samples the relative expression levels were used with either glyceraldehyde 3-phosphate dehydrogenase (GAPDH) or β-actin copy numbers as the reference. The PCR primers for GAPDH and β-actin were described previously [15,16]. The PCR primers for human PD-L1 and PD-L2 were adopted from previous reports [24,25].

### SDS/polyacrylamide gel electrophoresis and Western blot analysis

Cells were lysed in a lysis buffer (20 mM Tris-Base at pH 7.6, 150 mM NaCl, 1% NP-40, 10% glycerol) containing a cocktail of inhibitors, or subjected to Triton™ X-114 detergent-phase separation as described previously [13]. Triton™ X-114 extraction is a common method used to enrich for GPI-anchored membrane proteins [26], such as prostasin. Briefly, 5 × 10^5^ cells were lysed in 0.2 ml of lysis buffer prepared with TBS (10 mM Tris-HCl at pH 7.5 and 150 mM NaCl) containing 1% Triton™ X-114 and a protease inhibitor cocktail. The cells were extracted for 30 min at 4°C with gentle shaking, then spun at 12000×***g*** for 15 min to clear the debris. The Triton™ X-114 detergent was clouded out from the lysate supernatant during an incubation at 37°C for 3 min. The detergent phase (containing membrane-anchored proteins) was then separated from the aqueous phase (containing soluble proteins) via a brief centrifugation at 300×***g*** for 3 min at the room temperature. The clouding procedure was repeated to further purify the detergent phase free of the aqueous phase. The detergent phase was then resuspended in the original volume of TBS for further analysis. The total protein concentrations were determined using the Pierce™ BCA Protein Assay Kit (Thermo Fisher Scientific). Samples (20–40 micrograms per sample) were mixed with the sample buffer, resolved on polyacrylamide gels, transferred to a nitrocellulose membrane (Thermo Fisher Scientific), and blotted with the appropriate antibodies. To blot for prostasin and CD63, the samples were mixed with a sample buffer without reducing agents for the electrophoresis.

### Extracellular vesicle isolation, detergent-phase separation and protease activity binding assay

Equal number of cells (5 × 10^5^) were cultured and the conditioned media were collected for extracellular vesicle (EV) isolation. Three methods were carried out for EV isolation. First, the Invitrogen Total Exosome Isolation Reagent (Cat. No. 4478359) was used following the manufacturer’s protocol. Briefly, conditioned media were centrifuged at 2000×***g*** for 30 min to remove cell debris and large vesicles. The supernatant was then mixed with the reagent at 4°C overnight. The EV pellet was collected by centrifugation at 10000×***g*** for 1 h at 4°C. The pellet was washed briefly and gently with phosphate-buffered saline (PBS) once and resuspended in PBS at 1/10th of the starting medium volume. Second, polyethylene glycol (PEG, Thermo Fisher Scientific) was used as described [27] with modifications. Spun media were mixed with a PEG solution to reach a final concentration of 8.3%. The mixture was incubated at 4°C overnight and taken through the same centrifugation procedures described above. Third, the PEG-precipitated EVs were resuspended in PBS and subjected to ultracentrifugation at 100000×***g*** for 90 min to further eliminate protein carryover from the culture medium. The isolated EVs were subjected to the protease activity binding assay or detergent-phase separation [13], followed by analysis using SDS/polyacrylamide gel electrophoresis (PAGE) and Western blotting. The integrity of the isolated EVs was examined by Western blotting using antibodies against EV markers, i.e. CD63, Alix, Tsg101 and HSP70 [28,29] and by flow cytometry [30]. We did not find significant differences in the properties of EVs in the binding assay and the phase separation experiments using EVs isolated from the three different methods. We used both FBS-containing and FBS-free conditioned media for EV isolation and we observed similar results in our experiments.

### Flow cytometry

#### Cell surface labeling

Membrane-surface protein staining was performed according to suggestions from the manufacturers of the antibodies, or as described previously [31]. Briefly, cells were detached from culturing plates with 0.05% trypsin and 0.53 mM EDTA, washed with the growth medium containing 10% FBS and resuspended in the labeling buffer (PBS plus 10% FBS and 0.09% sodium azide). The PD-L1-APC or isotype-APC antibodies were added to the cell suspension at a ratio of 1 × 10^6^ cells per 5 µl of the antibody in 100 µl of the staining buffer. Cells were incubated on ice for 20 min in the dark, followed by washing in the labeling buffer twice, resuspending in fluorescence-activated cell sorting (FACS) buffer (PBS containing 0.2% BSA and 0.02% sodium azide) and flow cytometry analysis. For cell-surface staining of prostasin, the prostasin antibody was added into the cell suspension at a ratio of 1 × 10^6^ cells per 1 µl of the antibody in 100 µl of the staining buffer. Cells were incubated on ice for 30 min, washed with the labeling buffer twice, and then resuspended in the labeling buffer containing the Cy™2-labeled secondary antibody (at 1:100 dilution) for another 30 min on ice in the dark. For double antibody cell-surface staining, cells were labeled, in sequence, with the prostasin antibody, a goat anti-rabbit IgG-Cy2, and then PD-L1-APC or isotype-APC. Cells were washed in between the antibody staining steps, and then washed at the end of the staining, resuspended in the FACS buffer before flow cytometry analysis. A pre-immune rabbit serum was used as a control for cell-surface labeling of prostasin. A mock staining with the Cy™2-labeled secondary antibody alone (omitting the primary antibody) was also performed on cells as a control.

#### EV labeling

The isolated EVs were labeled with CellTrace™Violet (Cat. No. C34557, Thermo Fisher Scientific). The labeling procedure was performed as suggested by the manufacturer. Briefly, the isolated EVs were incubated with the CellTrace reagent at a final concentration of 5 µM for 20 min at 37°C. The mixture was diluted with PBS or the FACS buffer and subjected to flow cytometry analysis.

Flow cytometry was performed using a CytoFLEX S Flow Cytometer with the laser configuration of V2B2Y3R2 and operated by the CytExpert Software v2.2 (Beckman Coulter, Brea, CA). The violet side scatter (VSSC) detector configuration was set for the detection of EVs [30].

### Gene set enrichment analysis and pathway analysis

The RNA-seq Firehose data for lung squamous cell carcinoma (LUSC) and lung adenocarcinoma (LUAD) in The Cancer Genome Atlas (TCGA) Research Network (https://www.cancer.gov/tcga) were downloaded by using the Bioconductor package ‘RTCGAToolbox’ [32]. We extracted the normalized raw counts from the datasets with the FirehoseAnalyzeDates as ‘20160128’. An R script developed in-house classified the majority of the RNA-seq samples into three groups: samples with high PRSS8 expression (top 25%), high CD274 expression (top 25%) and low expression of both PRSS8 and CD274 (bottom 50% for both PRSS8 and CD274). The gene set enrichment analysis (GSEA) version 4.0.2 developed by the Broad Institute and the Hallmark gene sets from the Molecular Signatures Database (MSigDB) were utilized. Further comparisons of the enriched gene sets with <25% false discovery rate (FDR) for the PRSS8-high and CD274-high groups were performed and represented with Venn diagrams.

The rank ordered gene lists from GSEA with the ranking score threshold as 0.25 were further subjected to pathway analysis. Data were analyzed with the Ingenuity Pathway Analysis (IPA) developed by QIAGEN Inc. [33]. The hierarchical clustering heatmap of the identified pathways was generated with a z-score cutoff of 2.

### Statistical analysis

Data were expressed as mean ± standard deviations (SD). Student *t* test was used to compare the mean fluorescence intensities (MFIs) before and after treatments, in which, a *P*-value less than 0.05 is considered statistically significant. One-way analysis of variance (ANOVA) coupled with Tukey’s Honestly Significant Difference (HSD) post hoc test was used to determine statistical significance when comparing three or more independent groups, in which, a *P*-value less than 0.05 was considered statistically significant.

## Results

### Generation of Calu-3 human lung cancer cell sublines expressing prostasin variants or with prostasin knockout

**Figure 1:**
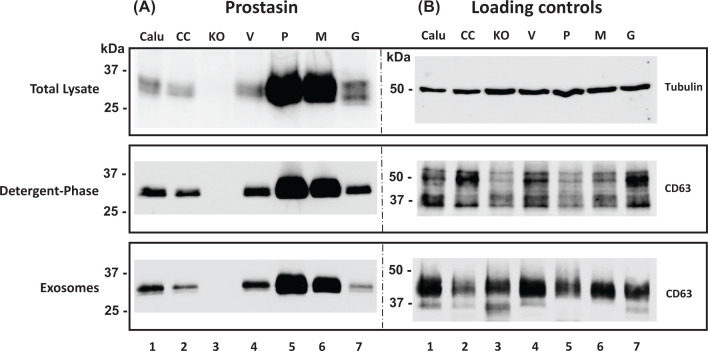
Western blot analysis of prostasin expression in Calu-3 sublines ** Top panel:** total cell lysate, 40 µg/lane; **middle panel:** Triton™ X-114-extracted membrane fractions from 40 µg of total cell lysate; **bottom panel:** isolated exosomes from 0.5 ml conditioned medium per sample (2.5 × 10^5^ cells). (**A**) Samples were immunoblotted with an antibody against human prostasin. (**B**) Samples were immunoblotted with antibodies against β-tubulin (total lysate) or CD63 (membrane fractions and exosomes) as loading controls. Calu: Calu-3 parent cells, CC: Calu-3 control subline with scrambled gRNA, KO: Calu-3 knockout subline with prostasin-specific gRNA, V: Calu-3 subline with vector alone, P: Calu-3 subline overexpressing the wildtype prostasin, M: Calu-3 subline overexpressing an active-site mutant prostasin, G: Calu-3 overexpressing an active prostasin without the GPI-anchor.

To determine the effect of prostasin expression changes on the expression of PD-L1, we established Calu-3 sublines overexpressing a wildtype human prostasin (P), an active-site mutant prostasin (M), a secreted active prostasin variant lacking the GPI-anchor (G), or the lentiviral vector-alone (V) as a control. We also established a Calu-3 subline with the prostasin gene knocked-out (KO) via CRISPR/Cas9 editing using a lentiviral vector and a control subline (CC) with a scrambled guide RNA. The differential prostasin/variant expression was ascertained in the Calu-3 sublines by Western blotting, and the results are shown in Figure 1A. The overexpressed prostasin protein can be readily detected in the sublines overexpressing the wildtype prostasin (P, Lane 5) or the protease-dead mutant prostasin (M, Lane 6). The subline overexpressing the secreted prostasin (G, Lane 7) has a moderate level of the prostasin protein in the cell lysate. Prostasin protein expression was not detected in the subline with the prostasin gene knocked out (KO, Lane 3), while baseline levels were detected in the parent Calu-3 cells (Lane 1) and in the vector control sublines (CC and V, Lanes 2 and 4).

The P and M forms of prostasin could be extracted from the cell membrane by means of Triton™ X-114 detergent-phase separation [13], confirming their membrane anchorage (Figure 1A, middle panel). As GPI-anchored proteins can be released from cells in the form of EVs [34,35], we analyzed and showed in Figure 1A (bottom panel) the presence of prostasin in the isolated EVs. In Figure 1B, the CD63 protein was used as a marker for the Triton™ X-114-extracted membrane fractions and for the isolated EV fractions. The amount of β-tubulin protein was used as a loading control for the total cell lysate analyzed in each sample.

### Prostasin potentiates IFNγ-induced PD-L1 expression in the Calu-3 lung cancer cells

**Figure 2:**
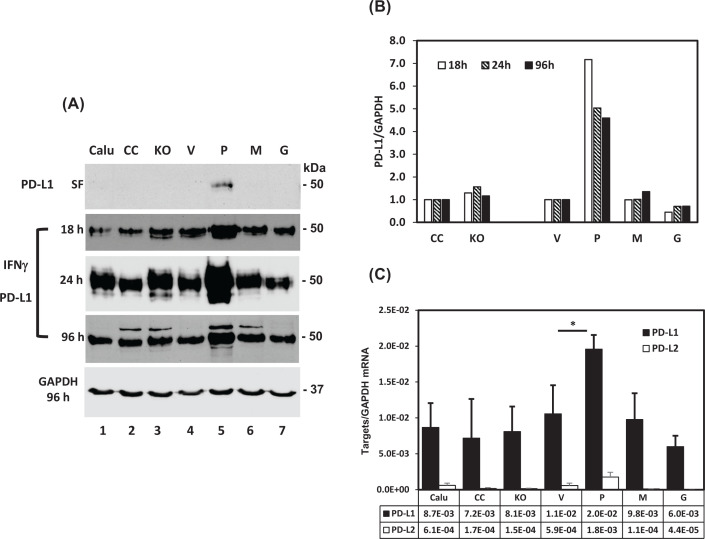
Prostasin increases PD-L1 expression (**A**) Western blot analysis of PD-L1 expression in Calu-3 sublines in serum-free cultures (SF) or treated with IFNγ at 100 ng/ml for various times as indicated. (**B**) Bar graph of IFNγ-induced PD-L1 expression in Calu-3 sublines normalized against GAPDH expression at various times shown in (A). (**C**) RT-qPCR analysis of PD-L1 and PD-L2 expression normalized against the GAPDH expression. Data are presented as mean ± SD (*n*=3). ANOVA *P*<0.05, * denotes *P*<0.05 as compared with the vector control (V). Abbreviation: RT-qPCR, real-time quantitative polymerase chain reaction.

Inflammatory cytokines such as IFNγ can induce PD-L1 expression [36,37], while prostasin is a negative regulator of IFNγ expression [15]. With the Calu-3 sublines expressing various functional forms of prostasin we investigated the expression of PD-L1 with or without IFNγ stimulation. The Calu-3 cells do not express detectable amounts of PD-L1, but an overexpression of the wildtype prostasin in the Calu-3 cells induced PD-L1 protein expression without any IFNγ treatment (Figure 2A, top panel, Lane 5). Similar results were also observed with normal human lung epithelial cells (Beas-2B) when prostasin was constitutively overexpressed (Supplementary Figure S1A, Lanes 2 and 5) and with normal human trophoblast cells (B6Tert-1) (Supplementary Figure S1B, Lane 5) when prostasin overexpression was induced by tetracycline (tet). The Beas-2B and B6Tert-1 cells express readily detectable endogenous PD-L1, which was enhanced by the overexpression of the wildtype prostasin. The active-site mutant prostasin (M) and the secreted active prostasin lacking the GPI-anchor (G) were unable to induce PD-L1 expression in the Calu-3 cells (Figure 2A, top panel, Lanes 6 and 7).

The PD-L1 protein became readily detected in all the cell types when treated with IFNγ, as shown in Figure 2A (middle three panels). The increased PD-L1 protein expression in the treated cells lasted for at least 96 h after the IFNγ stimulation (Figure 2B), indicating a low turnover rate [38,39]. PD-L1 expression was further increased in the Calu-3 subline overexpressing the wildtype prostasin as a GPI-anchored active membrane protease (Lane 5). But this enhancement was not observed in the sublines expressing either the inactive (M, Lane 6), or the GPI-anchor-free prostasin (G, Lane 7). Along with the up-regulation of the PD-L1 protein, the PD-L1 mRNA expression was also up-regulated by the IFNγ treatment, and was further increased only in the Calu-3P subline (Figure 2C, filled bars). The mRNA of another PD-1 ligand, PD-L2 (PDCD1LG2, programmed cell death 1 ligand 2) was also up-regulated by the IFNγ treatment (Figure 2C, unfilled bars), but the overall expression level was very low. PD-L2 protein expression was not detected by means of Western blotting under any experimental conditions.

We evaluated PD-L1 expression in the Calu-3 sublines cultured in Transwell air–liquid interface conditions to mimic the physiological context of lung epithelial cells and to allow the Calu-3 lung cells to differentiate before the IFNγ treatment [17]. For air–liquid Transwell cultures, the differentiation state of the Calu-3 cells was verified by confirming a high transepithelial electrical resistance (TEER > 1000 Ω-cm^2^) measured with an Epithelial Volt/Ohm Meter (EVOM). We observed that prostasin is able to induce PD-L1 mRNA expression in the air–liquid cultures (Supplementary Figure S1C).

### The IFNγ-induced overexpressed PD-L1 protein is localized on the surface of Calu-3 cells with prostasin

**Figure 3:**
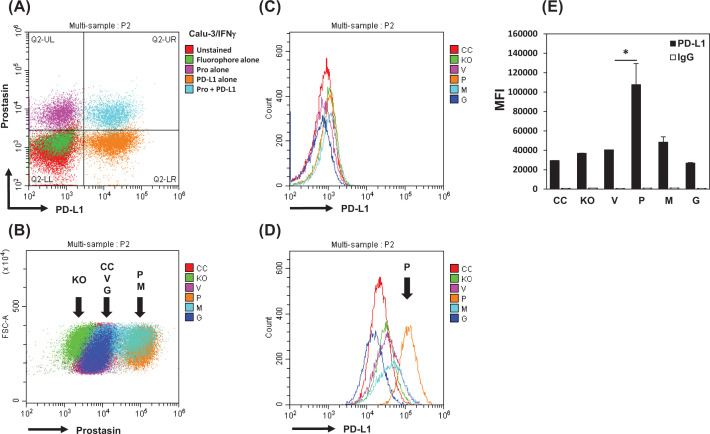
Flow cytometry analysis of PD-L1 and prostasin expressions in Calu-3 sublines (**A**) Representative dot plot of PD-L1 (x-axis) and prostasin expression (y-axis) in Calu-3 cells treated with IFNγ (100 ng/ml for 24 h). Red: unstained cells. Green: secondary antibody (anti-rabbit-Cy2) and isotype IgG-APC. Magenta: prostasin antibody-labeled cells. Orange: PD-L1-APC antibody-labeled cells. Sky blue: prostasin and PD-L1 antibodies double-labeled cells. (**B**) Representative dot plot of prostasin expression in Calu-3 sublines. CC (red): Calu-3 control subline with scrambled gRNA, KO (green): Calu-3 knockout subline with prostasin-specific gRNA, V (magenta): Calu-3 subline with vector alone, P (orange): Calu-3 subline overexpressing the wildtype prostasin, M (sky blue): Calu-3 subline overexpressing an active-site mutant prostasin, G (blue): Calu-3 overexpressing an active prostasin without the GPI anchor. (**C**,**D**) Histogram of PD-L1 expression in Calu-3 sublines without IFNγ treatment (C) or with IFNγ treatment (D). (**E**) MFI of PD-L1 (filled boxes) and isotype antibody (open boxes) from duplicate settings were quantified and presented in bar graphs. Data are presented as mean ± SD. ANOVA *P*<0.05, * denotes *P*<0.05 as compared with the vector control (V).

We performed flow cytometry to determine if the induced PD-L1 protein was on the plasma membrane of live Calu-3 cells and to quantify the cell-surface PD-L1 expression. The cells were also analyzed for the cell-surface prostasin. We first analyzed the parent Calu-3 cells treated with IFNγ. The analysis was restricted to live-gated Calu-3 cells identified by propidium iodide staining to avoid artifacts caused by non-specific staining of dead or dying cells. Live cells were further gated by FSC/SSC to discriminate doublets/cell aggregates from singlets. Double-staining for PD-L1 and prostasin revealed a uniform co-expression of these proteins, as shown in Figure 3A (Q2-UR). The double-staining signals for PD-L1 and prostasin were clearly separated from the control signals (Q2-LL), as well as the signals of single-staining for either prostasin (Q2-UL) or PD-L1 (Q2-LR).

Analysis of the cell-surface prostasin expression in the Calu-3 sublines is shown in Figure 3B. The sublines overexpressing the wildtype active prostasin (P) or the inactive mutant (M) had the strongest expression levels, while the vector controls (V and CC) and the secreted prostasin subline had moderate expression levels that fell between the KO and P/M populations. The relative levels of prostasin protein on the cell surface determined by flow cytometry were similar to those observed in the Western blot analysis of the total cell lysate (Figure 1).

We then analyzed PD-L1 expression in the Calu-3 sublines. Before the IFNγ treatment, all sublines had a background staining (Figure 3C). After the IFNγ treatment, PD-L1 expression was dramatically increased in all sublines, but the increase was the highest in the subline expressing the wildtype active prostasin (Figure 3D, Peak P). Figure 3E shows the MFI for the cell-surface PD-L1 levels from duplicate wells of each subline, along with the signal from isotype control staining (IgG), which was low across the board.

###  EVs contain prostasin and PD-L1

**Figure 4:**
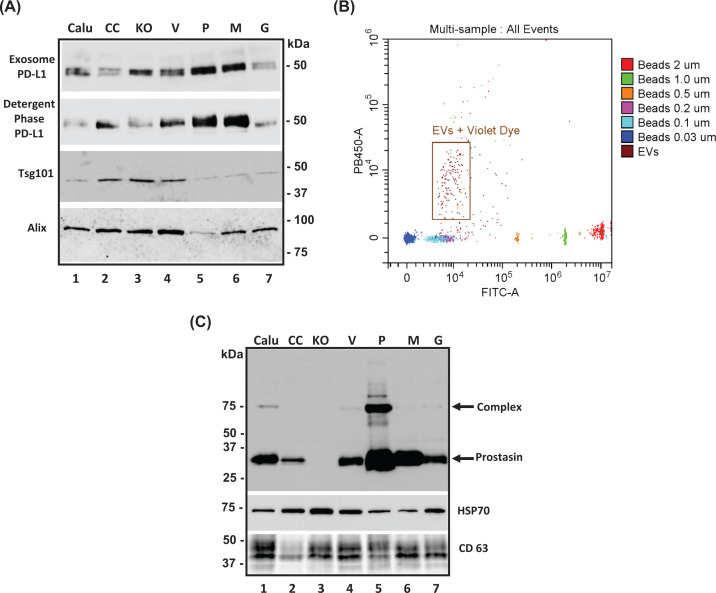
Identification of PD-L1 and active prostasin in exosomes (EVs) and membrane fractions (**A**) Western blot analysis of PD-L1 expression in exosomes (top panel) and membrane extractions (detergent phase). Exosome markers (Tsg101 and Alix) were blotted as loading controls. (**B**) Flow cytometry analysis of isolated exosomes (in brown box) labeled with a violet dye (PB450 channel) and sizing beads (0.03–2.0 µm) labeled with a green dye (FITC channel). (**C**) Western blot analysis of the binding assay of exosomes with purified PN-1 [43]. The wildtype prostasin in exosomes is active and able to form a covalent bound with its cognitive inhibitor PN-1 shifting the molecular weight of prostasin from 35 kDa (prostasin alone) to 75 kDa (prostasin and PN-1 complex). HSP70 and CD63 were blotted as loading controls for exosomes.

By means of Western blot analysis, we detected both PD-L1 (Figure 4A, top two panels) and prostasin (Figure 4C, top panel) in the EVs isolated from the conditioned media of IFNγ-treated Calu-3 sublines. The Tsg101, Alix, CD63 and HSP70 proteins were used as markers for the EVs [29]. Similar to prostasin (Figure 1), the PD-L1 protein can also be extracted and detected in the Triton™ X-114 detergent phase (Figure 4A, second panel from top), indicating a membrane anchorage in the EVs.

The EVs were labeled with CellTrace™Violet [40] for flow cytometry and were determined to be in the size range of 0.1–0.2 µm (Figure 4B, brown box) using green-fluorescent non-violet microsphere beads as the sizing reference. The active esterases inside the exosomes would cleave and convert the non-fluorescent violet dye into the fluorescent violet dye, which subsequently reacts with amine-containing proteins in the exosomes. The covalently bonded fluorescent dye–protein adducts were then detected in the PB450 violet channel in flow cytometry. Internalization of the membrane-permeable violet dye indicated that the membranous structures were maintained in the exosomes, and they were within the expected sizes [41,42].

The protease activity of prostasin in the EVs was ascertained by an established binding assay [13,43]. When incubated with the cognate prostasin inhibitor protease nexin-1 (PN-1), a higher molecular weight complex was formed via the binding of the prostasin active-site serine to the suicide substrate inhibitor PN-1 (Figure 4C, top panel). The overexpressed wildtype prostasin (P) in the EVs showed a strong binding activity, while the inactive mutant prostasin (M) had no binding activity. The low-level endogenous prostasin in Calu-3 and the other sublines exhibited a basal binding activity, except in the KO subline with the prostasin gene knocked out.

### Prostasin increases PD-L1 expression via the EGF-EGFR axis

**Figure 5:**
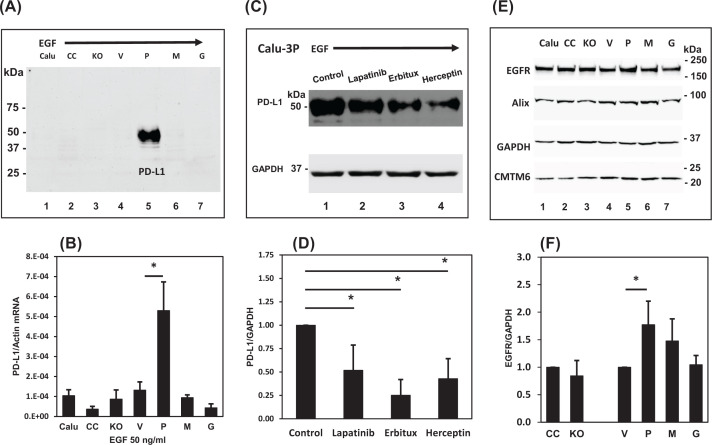
Prostasin up-regulates PD-L1 expression via the EGFR signaling pathway (**A**) Immunoblotting of PD-L1 induced by EGF (50 ng/ml for 24 h) in Calu-3 sublines. (**B**) RT-qPCR analysis of PD-L1 expression in Calu-3 sublines treated with EGF (50 ng/ml, *n*=3, ANOVA *P*<0.05, * denotes *P*<0.05 as compared with the vector control (V). (**C**) Top panel, EGF-induced PD-L1 expression in Calu-3P can be inhibited by an EGFR kinase inhibitor (Lapatinib, 0.2 µM) and antibodies against EGFR (Erbitux, 40 µg/ml) and Her-2 (Herceptin, 40 µg/ml); bottom panel, immunoblotting of GAPDH as a loading control. (**D**) Bar graph of (C) (*n*=4), * denotes *P*<0.05 as compared with the control. (**E**) Immunoblotting of EGFR, Alix, CMTM6 and GAPDH. (**F**) Bar graph of EGFR in (E) (*n*=3), * denotes *P*<0.05, as compared with the vector control (V). Abbreviation: RT-qPCR, real-time quantitative polymerase chain reaction.

To investigate the signaling pathways involved in the prostasin-mediated PD-L1 up-regulation, we first assessed the role of the epidermal growth factor-epidermal growth factor receptor (EGF-EGFR) pathway. Previously, we showed that prostasin regulates EGFR activity [23,44] and others have shown that the activated EGFR promotes PD-L1 expression [45]. We show in Figure 5A that activation of the EGF-EGFR axis by EGF up-regulated PD-L1 expression only in the subline overexpressing the wildtype prostasin (Calu-3P, Lane 5). This up-regulation was also observed at the mRNA level (Figure 5B), indicating a transcriptional mechanism.

The Calu-3 cells express ErbB1/EGFR and ErbB2/Her2, and are sensitive to EGFR TKIs and the EGFR-targeting antibody cetuximab/Erbitux [46–48]. We show in Figure 5C,D that the EGF-induced PD-L1 expression in the Calu-3P subline (Lane 1) can be partially blocked by the dual-EGFR/Her2 TKI lapatinib (Lane 2), cetuximab/Erbitux (Lane 3), and the Her2-targeting antibody trastuzumab/Herceptin (Lane 4). The programmed cell death 6-interacting protein (Alix) and the chemokine-like factor-like (CKLF) MARVEL transmembrane domain containing family member 6 (CMTM6) protein are also involved in the up-regulation of PD-L1 [49–51]. But as shown in Figure 5E, we did not observe significant changes of expression for either Alix or CMTM6 in the Calu-3 sublines treated with EGF. We observed an increased EGFR expression in the Calu-3P cells (Figure 5E, Lane 5, and Figure 5F).

### The PKC and MAPK pathways contribute to prostasin-induced PD-L1 expression

**Figure 6:**
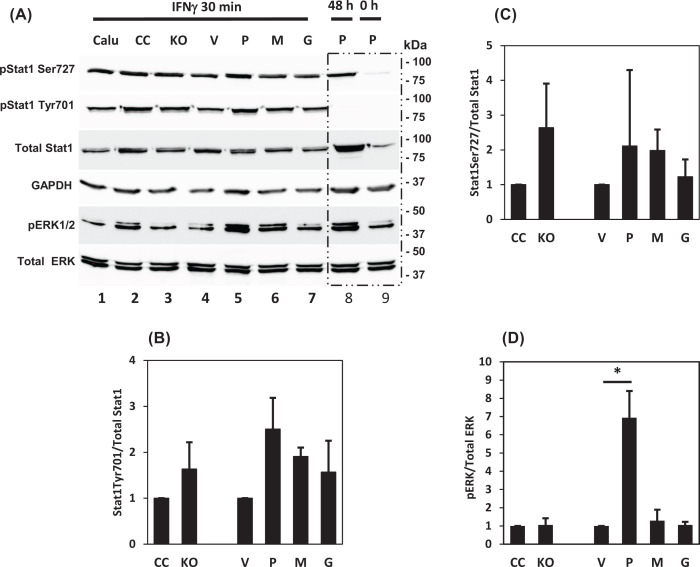
Prostasin activation of MAPK pathway manifested by ERK1/2 phosphorylation (**A**) Representative images of Western blot analysis on Calu-3 sublines treated with 100 ng/ml IFNγ for 30 min. Calu-3P cells without the treatment (0 h) and with the treatment for 48 h were used for monitoring protein phosphorylation as indicated. Both pStat1Tyr^701^ and pStat1Ser^727^ were increased upon IFNγ treatment, while phosphorylation of Stat1Ser^727^ sustained for at least 48 h. (**B–D**) Bar graphs of phosphorylated proteins in (A), * denotes *P*<0.05, as compared with the vector control (V).

The Janus kinase/signal transducer and activator of transcription 1 (JAK/STAT1) signaling pathway is a major regulator of IFNγ-induced PD-L1 expression in many cell lines and patient samples [7,52]. In Figure 6A, we show that Stat1 was phosphorylated at both Ser^727^ and Tyr^701^ in the IFNγ-treated cells. The phosphorylation at Tyr^701^ diminished within 48 h, but the phosphorylation at Ser^727^ remained after 48 h. No statistically significant changes of Stat1 phosphorylation was observed across the different cell types (Figure 6B,C). On the other hand, phosphorylation of the extracellular signal-regulated kinase 1/2 (pERK1/2) was the highest in the cells overexpressing the wildtype prostasin (Figure 6A, Lane 5; Figure 6D).

**Figure 7:**
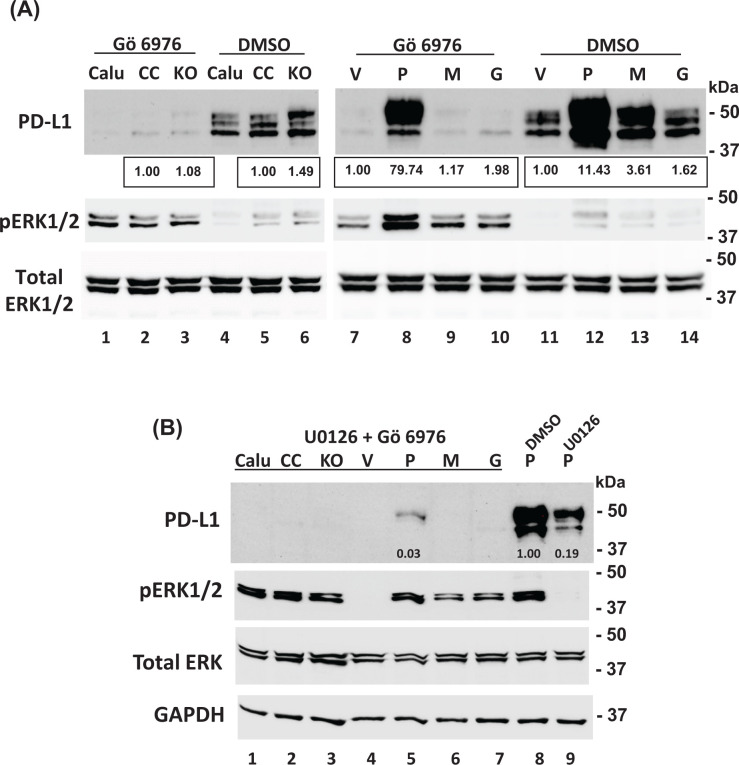
Prostasin up-regulates PD-L1 expression via activation of MAPK/ERK pathways (**A**) Cells were treated with the PKCα inhibitor Gö 6976 (4 µM) for 30 min followed by IFNγ treatment (100 ng/ml) for 7 h. Thirty micrograms of total cell lysates from each Calu-3 subline was subjected to Western blot analysis with antibodies as indicated. Inhibition of PKCα increased phosphorylated ERK1/2 content in Calu-3P (lane 8) and sustained the expression of PD-L1 (lane 8). (**B**) Cells were analyzed as described in (A), except cells were treated with an MEK inhibitor (U0126, 5 µM) for 15 min, the PKCα inhibitor Gö 6976 (4 µM) for 45 min and then IFNγ (100 ng/ml) for 7 h. DMSO was used as the vehicle control for the inhibitors. Inhibition of both PKCα and ERK1/2 phosphorylation further reduced PD-L1 expression. Relative densities of the PD-L1 bands normalized against GAPDH are indicated under each band.

The mechanism of PD-L1 expression regulation by IFNγ is complex. An initial screening of a panel of inhibitors identified the protein kinase C (PKC) inhibitor Gö 6976 as being able to attenuate PD-L1 expression in the prostasin overexpressing cells treated with IFNγ. PKC can be activated by diacylglycerol (DAG), a product of the IFNγ-activated phospholipase C γ 2 (PLCγ-2) [53]. In Figure 7A, we show that inhibition of PKCα by Gö 6976 greatly reduced PD-L1 expression in all Calu-3 sublines treated with IFNγ, but the expression of PD-L1 remained high only in the Calu-3P cells (top panel, Lane 8). The PKCα inhibitor was shown to have promoted ERK phosphorylation (pERK1/2) in the Calu-3P cells (Figure 7A, middle panel, Lane 8). The MEK inhibitor U0126 further inhibited the IFNγ-induced PD-L1 expression in these cells, synergistically with the PKCα inhibition by Gö 6976, as shown in Figure 7B.

**Figure 8:**
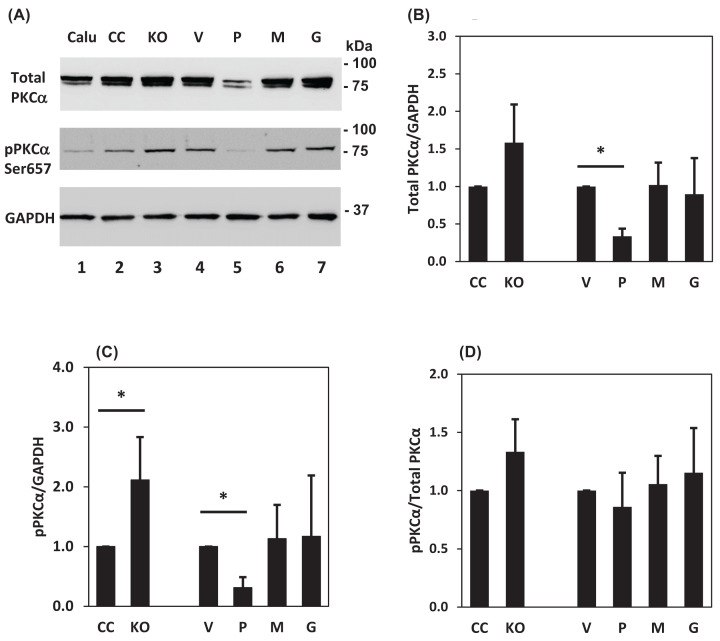
Prostasin down-regulates PKCα expression Thirty micrograms of total cell lysates from each Calu-3 subline was subjected to Western blot analysis. (**A**) Reduced PKCα expression is seen only in samples overexpressing the wildtype membrane-anchored prostasin (lane 5). (**B–D**) Results of four independent experiments were analyzed, quantified and presented as bar graphs: total PKCα normalized against GAPDH (B); phosphorylated PKCα normalized against GAPDH (C); ratio of phosphorylated PKCα in total PKCα content (D). Data are presented as mean ± SD, * denotes *P*<0.05, as compared with the controls (CC or V).

We evaluated the status of the PKCα protein in the Calu-3 sublines and show that the prostasin overexpression reduced the level of total PKCα in the Calu-3P cells (Figure 8A,B), with a corresponding reduction in the phosphorylation of Ser^657^ in PKCα (Figure 8C). We have also observed a PKCα down-regulation by prostasin re-expression in human prostate cancer cell lines PC-3 and DU-145 (Supplementary Figure S2). The Calu-3 subline with the prostasin gene knockout (KO) had a significant increase in the phosphorylation of Ser^657^ in PKCα (Figure 8C).

### The GSEA

**Figure 9:**
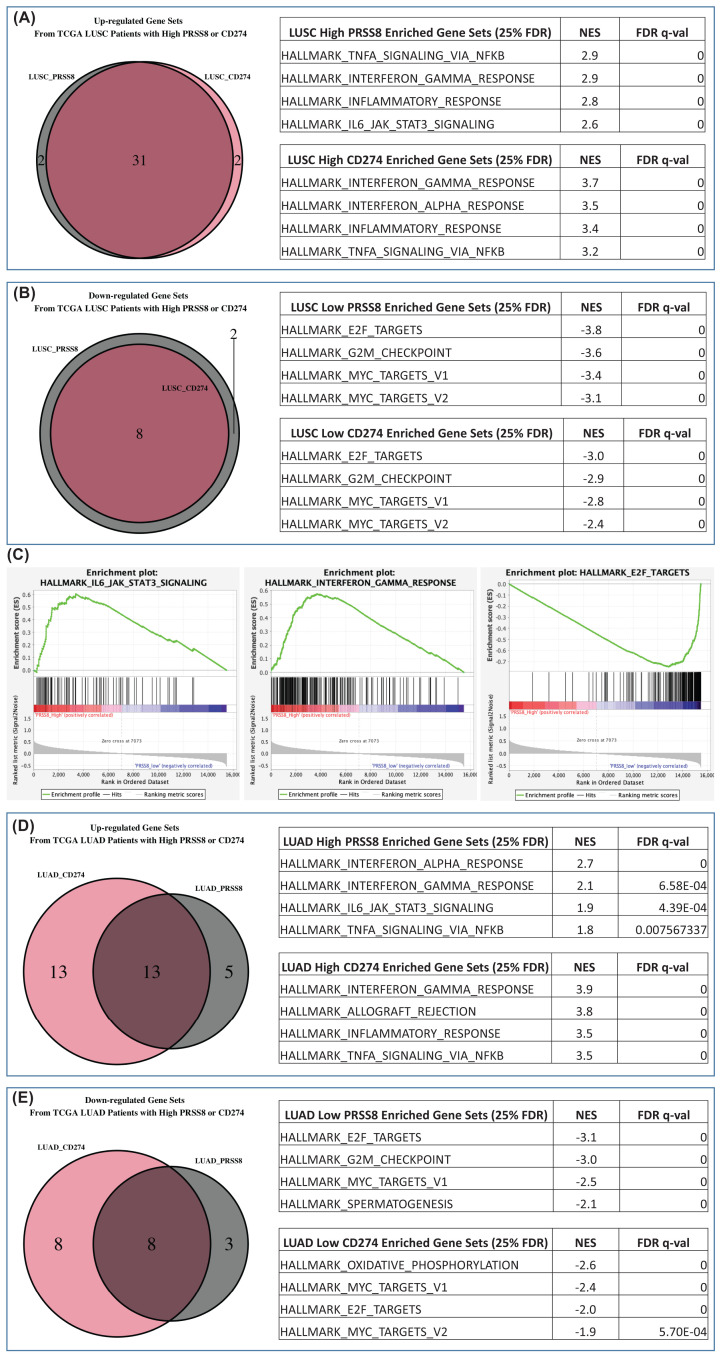
GSEA of LUSC and LUAD from TCGA The selected top four gene sets are shown (LUSC, A-C; LUAD, D,E). The complete list of enriched gene sets is in Supplementary Figure S3A–H. (A) Thirty-one out of thirty-three enriched gene sets from the samples with high CD274 expression were shared with the enriched gene sets from samples with high PRSS8 expression. (B) Eight out of eight enriched gene sets from samples with low CD274 expression were shared with the enriched gene sets from samples with low PRSS8 expression. (C) The hallmark gene sets from the MSigDB gene sets were used. The hallmark IL6_JAK_Stat3 signaling and IFNγ response are listed on the top shared enriched gene sets for both PRSS8 and CD274 high-expression sample groups. The E2F_targets is listed on the top shared enriched gene sets for both PRSS8 and CD274 low-expression sample groups. (D) Thirteen out of eighteen enriched gene sets from the samples with high PRSS8 expression were shared with the enriched gene sets from the samples with high CD274 expression. (E) Eight out of eleven enriched gene sets from samples with low PRSS8 expression were shared with the enriched gene sets from the samples with low CD274 expression. All significant gene sets are defined with 25% FDR as the threshold.

To explore the functional relationships of prostasin (PRSS8) and PD-L1 (CD274), we performed GSEA to identify enriched gene sets in PRSS8-high (top 25%) or CD274-high (top 25%) patients with LUSC. The normalized read counts of RNA-seq data were retrieved from the TCGA database [54]. We defined a patient group with a low expression (bottom 50%) of both PRSS8 and PD-L1 as the control group in the GSEA. The hypothesis that PRSS8 and CD274 may be involved in some common pathways can be supported if we observe overlapping enriched gene sets. The GSEA results with the LUSC datasets have shown that the enriched gene sets from the PRSS8-high group and the CD274-high group are substantially overlapped (Figure 9A). A total of 33 functional gene clusters were up-regulated in the CD274-high group, and among them, 31 matched the up-regulated gene sets in the PRSS8-high group. Likewise, the down-regulated gene clusters in the PRSS8-high and CD274-high groups showed a significant overlap as well (Figure 9B). All eight down-regulated gene sets from the CD274-high group were found on the down-regulated list from the PRSS8-high group.

The common enriched gene sets suggest multiple shared signaling pathways in the patient groups with high PRSS8 or high CD274 expression. These include the IL6_JAK_Stat3 signaling and IFNγ response pathways (Figure 9C). In addition, a hierarchical clustering heatmap of pathways identified by using the IPA revealed that the ERK/MAPK signaling is shared by the PRSS8-high and the CD274-high groups (Supplementary Figure S4). These findings support our experimental results that PRSS8 is involved in the MAPK pathway. To further test the potential clinical relevance of the relationship between prostasin and PD-L1 we performed GSEA for LUAD patients (Figure 9D,E). The same common gene enrichment patterns were observed for the LUAD tumors with high expression for both PRSS8 and CD274 among the multiple signaling pathways identified in the LUSC tumors.

## Discussion

PD-L1 is a major player in tumor cell evasion of immune surveillance and has been exploited as a target and a marker for cancer immunotherapy. In epithelial cancers, tumor cell PD-L1 expression is a critical factor of consideration for achieving and improving efficacy. It had been well established that the inflammatory cytokines, such as IFNγ, abundant in the tumor microenvironment, boost tumor cell PD-L1 expression. The membrane-associated extracellular serine protease prostasin is a major player in epithelial homeostasis and has a role in regulating the innate immune response and the expression of inflammatory cytokines, including IFNγ [15]. Prostasin is also involved in transcriptional and post-translational regulation of membrane proteins, in particular, growth factor receptors such as EGFR, and receptors involved in cytokine production, such as the toll-like receptor 4 (TLR4) [55]. In this study, we aimed to determine if there is a cross-talk between the cell signaling pathways regulated by prostasin and the mechanisms that regulate PD-L1 expression.

Using human NSCLC cell line Calu-3 sublines expressing various forms of prostasin or with a prostasin gene knockout, we first demonstrated the responsiveness of the PD-L1 gene to the prostasin overexpression. Without any external stimuli, the PD-L1 protein expression was induced from a null background by the wildtype prostasin, but not the protease-dead or the membrane-anchor-free, secreted prostasin variant (Figure 2). This result suggests that the prostasin-mediated up-regulation of PD-L1 requires its serine protease function, as well as the membrane anchorage. We then employed a known positive regulator of PD-L1 expression, IFNγ, to investigate if the membrane-associated prostasin could have an impact on an IFNγ induction of PD-L1 expression. Indeed, the IFNγ up-regulation of PD-L1 was greatly enhanced, also by only the wildtype prostasin and involved a transcriptional mechanism (Figure 2). In this context, an outside-in mechanism of signal intervention is postulated for prostasin, likely involving its interactions with relevant membrane proteins, i.e., growth factor receptors and cytokine receptors.

Our focus turned to the MAPK signaling pathway at first, because an activation of this pathway stabilizes the PD-L1 mRNA [56] and prostasin regulates a direct upstream growth factor receptor, EGFR [23,44]. We stimulated the cells with EGF, and a robust transcriptional up-regulation of PD-L1 was observed in the cells overexpressing the wildtype prostasin (Figure 5). The use of EGF independently of IFNγ allowed us to tease out the involvement of the MAPK signaling pathway. On the other hand, we did not observe a statistically significant change in the phosphorylation of Stat1 (Figure 6), a signal relay downstream of the IFNγ receptors. Alternatively, IFNγ signaling can activate PLCγ-2 [53], which hydrolyzes phosphatidylinositol (3,4,5)-trisphosphate (PIP3) and generates DAG to activate protein kinase C (PKC). The PKCα inhibitor Gö 6976 was able to tame the IFNγ induction of PD-L1 expression, but the effect was incomplete in the cells expressing the wildtype prostasin (Figure 7A). A synergistic suppression of IFNγ induction of PD-L1 was observed when the MEK inhibitor U0126 was used in combination with Gö 6976 (Figure 7B). PKCα has been shown to regulate EGFR activation and internalization [57,58]. Herein we showed that PKCα was significantly down-regulated by the wildtype prostasin (Figure 8). We postulate that the down-regulation of PKCα by prostasin could reduce EGFR internalization and ubiquitination [59], contributing to the increased PD-L1 expression in response to IFNγ via such a cross-talk to the EGFR signaling pathway. In support of this, the EGFR level in the Calu-3P subline expressing the wildtype prostasin was increased (Figure 5F).

Physiologically, prostasin protects the integrity of the epithelium by down-regulating inflammatory cytokine production, e.g., IFNγ [15,16] and by enhancing tight junction formation [17]. The endogenously expressed PD-L1 in normal epithelial cells can be viewed as a mechanism for increasing the tolerance of normal cells during an immune attack or for preventing damage caused by an overly aggressive local inflammatory response. In tumors, PD-L1 expression is up-regulated by IFNγ and other cytokines in the tumor microenvironment. The expression of PD-L1 could be further enhanced and sustained by the presence of prostasin in tumor cells. It is possible that the co-expression of prostasin in PD-L1-positive tumor cells may sensitize the cells to the anti-PD-L1 antibodies in immunotherapy targeting the PD-1/PD-L1 checkpoint. Indeed, reports have shown that higher PD-L1 staining in tumor cells is associated with a higher response rate and improved efficacy in PD-1/PD-L1 blockade therapy [60,61]. Whether PD-L1 expression is up-regulated in prostasin-positive tumor cells in patients would then warrant further investigation.

An interrogation into the TCGA database revealed that prostasin (PRSS8) and PD-L1 (CD274) are involved in common pathways in both LUSC and LUAD (Figure 9). The IL6_JAK_Stat3 signaling and IFNγ response pathways are shared in PRSS8-high and CD274-high patients. It is possible that prostasin participates in the IL-6_JAK-Stat3 pathway via activation of the MAPK pathway as it cross-talks with the IL-6-JAK/STAT pathway [62]. We have also observed shared pathways in both LUSC and LUAD patient groups with low PRSS8 expression and low CD274 expression. The E2F_targets related pathway is among the shared pathways in the low expressors. This finding is consistent with our result that PKCα was down-regulated by PRSS8 overexpression, as PKC activation increases E2F-1 expression [63].

PD-L1 is released by metastatic melanoma and glioblastoma cancer cells in EVs, though the physiological outcome or significance remains unclear. The dynamically changing amounts of circulating exosomes carrying PD-L1 in patients was suggested as a predictor for anti-PD-1 therapy [64,65]. In addition, antibodies can be neutralized in patient blood by the corresponding antigen in the exosomes released by cancer cells during an antibody-based immunotherapy. This competition can result in a reduced treatment efficacy, as in the case of rituximab for treating lymphoma [66]. In this study, we showed that the protease-active prostasin and PD-L1 co-localized in the EVs released by lung cancer cells. It will be interesting to learn in future studies if the active prostasin in the EVs could interfere with the PD-L1 function in immune surveillance or immune editing.

## Conclusion

**Figure 10:**
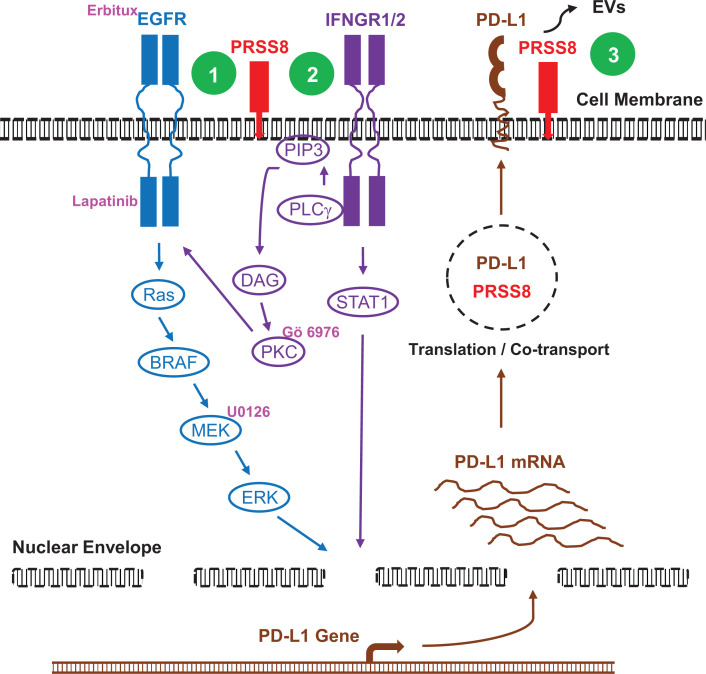
Signaling pathways in prostasin regulation of PD-L1 expression **(Green) Node 1:** EGFR signaling, with Erbitux and lapatinib showing effects on prostasin regulation of PD-L1 expression. **Node 2:** IFNγ signaling via PKCα, with Gö 6976 and U0126 showing effects on the prostasin-mediated potentiation of PD-L1 induction by IFNγ. **Node 3:** Prostasin and PD-L1 co-localization in exosomes.

Prostasin is identified as a potent regulator of PD-L1 expression induced by the inflammatory cytokine IFNγ in human lung epithelial and cancer cells. This action requires the serine protease activity and the membrane anchorage and is mediated by the cross-talk between IFNγ signaling and EGF-EGFR signaling, involving PKCα, as illustrated by Nodes 1 and 2 in Figure 10. Prostasin and PD-L1 co-localize in exosomes shed from lung epithelial or cancer cells, as illustrated by Node 3 in Figure 10. Understanding the roles played by prostasin in the tumor microenvironment could provide information on if and how prostasin can be explored and developed as therapeutics or a marker for immune editing.

## Supplementary Material

Supplementary Figures S1-S5

## Data Availability

The TCGA datasets of LUSC and LUAD (Level 3) used in current study are publicly available at the TCGA Research Network (https://www.cancer.gov/tcga).

## Open Access

Open access for this article was enabled by the participation of University of Central Florida in an all-inclusive *Read & Publish* pilot with Portland Press and the Biochemical Society under a transformative agreement with EBSCO.

## Abbreviations

APC: allophycocyanin
ATCC: American Type Culture Collection
B6Tert-1: human telomerase reverse transcriptase (hTERT)-immortalized normal human trophoblast cell line
Beas-2B: human normal lung epithelial cell line
Calu-3: human lung adenocarcinoma cell line
CD274: cluster of differentiation 274 (PD-L1)
CMTM6: CKLF-like MARVEL transmembrane domain containing protein 6
DAG: diacylglycerol
DU145: human prostate carcinoma cell line
EGF-EGFR: epidermal growth factor-epidermal growth factor receptor
EV: extracellular vesicle
FACS: fluorescence-activated cell sorting
FBS: fetal bovine serum
GAPDH: glyceraldehyde 3-phosphate dehydrogenase
GPI: glycosylphosphatidylinositol
GSEA: Gene Set Enrichment Analysis
IFNγ: interferon-γ
IPA: Ingenuity Pathway Analysis
JAK/STAT1: Janus kinase/signal transducer and activator of transcription 1
LUAD: lung adenocarcinoma
LUSC: lung squamous cell carcinoma
MAPK: mitogen activated protein kinase
MEK: Mitogen-activated protein kinase kinase
MFI: mean fluorescence intensity
NSCLC: non-small cell lung cancer
PBS: phosphate-buffered saline
PC-3: human prostate carcinoma cell line
PD-1: programmed cell death protein 1
PD-L1: programmed death-ligand 1
PEG: polyethylene glycol
pERK1/2: phosphorylated extracellular signal-regulated kinase
PKC: protein kinase C
PLCγ-2: phospholipase C γ 2
PN-1: protease nexin-1
PRSS8: serine protease 8 (prostasin)
TCGA: The Cancer Genome Atlas
TKI: tyrosine kinase inhibitor
