# Supplementary material for: Prostasin regulates PD-L1 expression in human lung cancer cells
Source: Biosci Rep. 2021 Jul 9;41(7):BSR20211370. doi: 10.1042/BSR20211370 (PMC8273379; doi:10.1042/BSR20211370)

Supplementary Figure 1

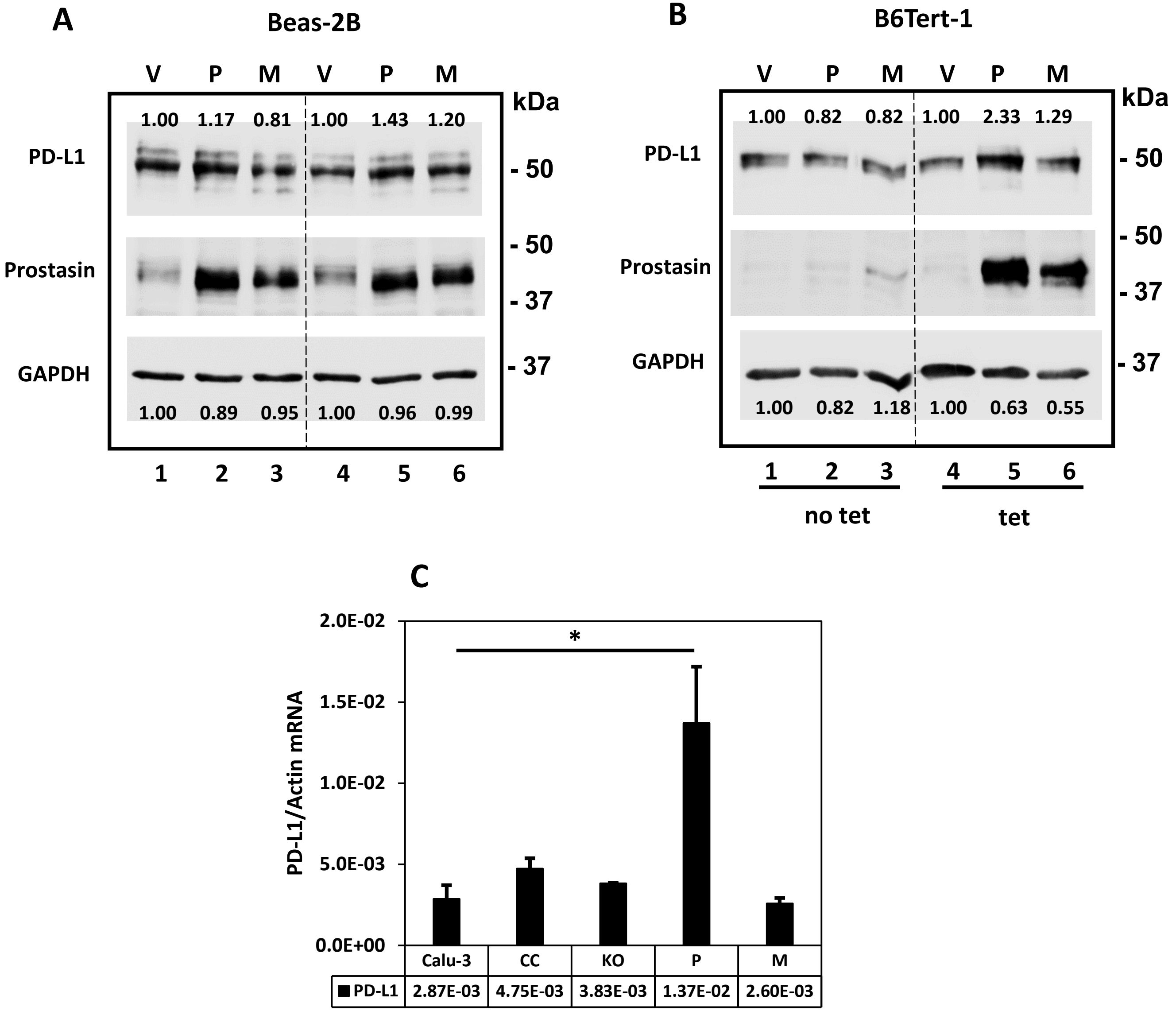

Supplementary Figure 2

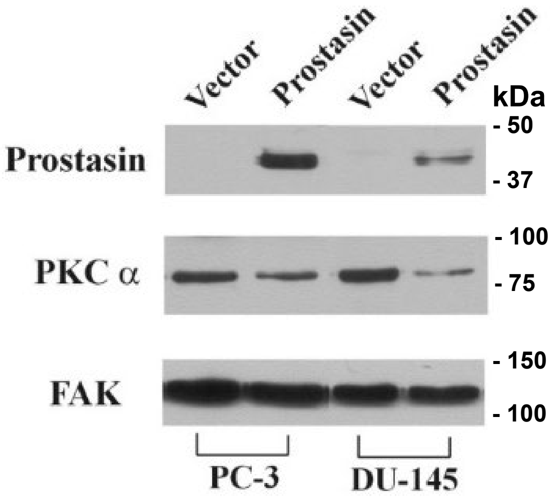

Supplementary Figure 3 (a-h)

a. LUSC High PRSS8 Enriched Gene Sets

LUSC High PRSS8 Enriched Gene Sets (25% FDR)	NES	FDR q-val
HALLMARK_TNFA_SIGNALING_VIA_NFKB	2.9	0
HALLMARK_INTERFERON_GAMMA_RESPONSE	2.9	0
HALLMARK_INFLAMMATORY_RESPONSE	2.8	0
HALLMARK_IL6_JAK_STAT3_SIGNALING	2.6	0
HALLMARK_COAGULATION	2.5	0
HALLMARK_COMPLEMENT	2.5	0
HALLMARK_INTERFERON_ALPHA_RESPONSE	2.5	0
HALLMARK_ALLOGRAFT_REJECTION	2.1	0
HALLMARK_IL2_STAT5_SIGNALING	2.1	0
HALLMARK_APICAL_JUNCTION	2.0	0
HALLMARK_TGF_BETA_SIGNALING	2.0	0
HALLMARK_MYOGENESIS	2.0	0
HALLMARK_ESTROGEN_RESPONSE_EARLY	2.0	0
HALLMARK_BILE_ACID_METABOLISM	2.0	0
HALLMARK_APOPTOSIS	2.0	0
HALLMARK_KRAS_SIGNALING_UP	1.9	0
HALLMARK_HYPOXIA	1.8	2.39E-04
HALLMARK_UV_RESPONSE_DN	1.8	3.81E-04
HALLMARK_EPITHELIAL_MESENCHYMAL_TRANSITION	1.8	0.001179557
HALLMARK_XENOBIOTIC_METABOLISM	1.8	0.001188436
HALLMARK_ANGIOGENESIS	1.8	0.001584159
HALLMARK_ADIPOGENESIS	1.7	0.001991312
HALLMARK_P53_PATHWAY	1.7	0.002751719
HALLMARK_HEME_METABOLISM	1.6	0.003917295
HALLMARK_PEROXISOME	1.5	0.009721883
HALLMARK_ESTROGEN_RESPONSE_LATE	1.5	0.011090398
HALLMARK_CHOLESTEROL_HOMEOSTASIS	1.5	0.012853715
HALLMARK_REACTIVE_OXYGEN_SPECIES_PATHWAY	1.4	0.021902649
HALLMARK_ANDROGEN_RESPONSE	1.4	0.022806939
HALLMARK_FATTY_ACID_METABOLISM	1.3	0.055563193
HALLMARK_UV_RESPONSE_UP	1.3	0.06734516
HALLMARK_APICAL_SURFACE	1.2	0.16197442
HALLMARK_KRAS_SIGNALING_DN	1.2	0.20365763

b. LUSC High CD274 Enriched Gene Sets

LUSC High CD274 Enriched Gene Sets (25% FDR)	NES	FDR q-val
HALLMARK_INTERFERON_GAMMA_RESPONSE	3.7	0
HALLMARK_INTERFERON_ALPHA_RESPONSE	3.5	0
HALLMARK_INFLAMMATORY_RESPONSE	3.4	0
HALLMARK_TNFA_SIGNALING_VIA_NFKB	3.2	0
HALLMARK_ALLOGRAFT_REJECTION	3.1	0
HALLMARK_COMPLEMENT	3.0	0
HALLMARK_IL6_JAK_STAT3_SIGNALING	2.9	0
HALLMARK_IL2_STAT5_SIGNALING	2.7	0
HALLMARK_COAGULATION	2.3	0
HALLMARK_KRAS_SIGNALING_UP	2.3	0
HALLMARK_APOPTOSIS	2.2	0
HALLMARK_REACTIVE_OXYGEN_SPECIES_PATHWAY	2.0	2.23E-04
HALLMARK_HYPOXIA	1.9	2.06E-04
HALLMARK_XENOBIOTIC_METABOLISM	1.9	6.14E-04
HALLMARK_HEME_METABOLISM	1.8	6.55E-04
HALLMARK_TGF_BETA_SIGNALING	1.8	7.09E-04
HALLMARK_MYOGENESIS	1.8	8.09E-04
HALLMARK_UV_RESPONSE_DN	1.8	9.02E-04
HALLMARK_BILE_ACID_METABOLISM	1.8	0.001168116
HALLMARK_APICAL_JUNCTION	1.8	0.001406594
HALLMARK_UV_RESPONSE_UP	1.7	0.002580947
HALLMARK_ESTROGEN_RESPONSE_EARLY	1.7	0.003043273
HALLMARK_EPITHELIAL_MESENCHYMAL_TRANSITION	1.6	0.009378419
HALLMARK_ADIPOGENESIS	1.5	0.012899715
HALLMARK_P53_PATHWAY	1.5	0.020130154
HALLMARK_ANDROGEN_RESPONSE	1.4	0.038793676
HALLMARK_FATTY_ACID_METABOLISM	1.4	0.03879502
HALLMARK_APICAL_SURFACE	1.4	0.038254187
HALLMARK_PROTEIN_SECRETION	1.4	0.050922032
HALLMARK_ESTROGEN_RESPONSE_LATE	1.3	0.07497506
HALLMARK_KRAS_SIGNALING_DN	1.2	0.14584666
HALLMARK_CHOLESTEROL_HOMEOSTASIS	1.2	0.1506702
HALLMARK_PI3K_AKT_MTOR_SIGNALING	1.2	0.19768856

c. LUSC Low PRSS8 Enriched Gene Sets

LUSC Low PRSS8 Enriched Gene Sets (25% FDR)	NES	FDR q-val
HALLMARK_E2F_TARGETS	-3.8	0
HALLMARK_G2M_CHECKPOINT	-3.6	0
HALLMARK_MYC_TARGETS_V1	-3.4	0
HALLMARK_MYC_TARGETS_V2	-3.1	0
HALLMARK_MITOTIC_SPINDLE	-2.1	1.04E-04
HALLMARK_SPERMATOGENESIS	-2.0	8.70E-05
HALLMARK_MTORC1_SIGNALING	-1.9	1.60E-04
HALLMARK_OXIDATIVE_PHOSPHORYLATION	-1.8	4.34E-04
HALLMARK_DNA_REPAIR	-1.8	3.86E-04
HALLMARK_UNFOLDED_PROTEIN_RESPONSE	-1.7	0.001982023

d. LUSC Low CD274 Enriched Gene Sets

LUSC Low CD274 Enriched Gene Sets (25% FDR)	NES	FDR q-val
HALLMARK_E2F_TARGETS	-3.0	0
HALLMARK_G2M_CHECKPOINT	-2.9	0
HALLMARK_MYC_TARGETS_V1	-2.8	0
HALLMARK_MYC_TARGETS_V2	-2.4	0
HALLMARK_OXIDATIVE_PHOSPHORYLATION	-1.9	9.45E-04
HALLMARK_DNA_REPAIR	-1.8	0.00147399
HALLMARK_SPERMATOGENESIS	-1.8	0.001415876
HALLMARK_MITOTIC_SPINDLE	-1.3	0.060848035

e. LUAD High PRSS8 Enriched Gene Sets

LUAD High PRSS8 Enriched Gene Sets (25% FDR)	NES	FDR q-val
HALLMARK_INTERFERON_ALPHA_RESPONSE	2.7	0
HALLMARK_INTERFERON_GAMMA_RESPONSE	2.1	6.58E-04
HALLMARK_IL6_JAK_STAT3_SIGNALING	1.9	4.39E-04
HALLMARK_TNFA_SIGNALING_VIA_NFKB	1.8	0.007567337
HALLMARK_P53_PATHWAY	1.7	0.007077679
HALLMARK_INFLAMMATORY_RESPONSE	1.7	0.009387618
HALLMARK_APICAL_JUNCTION	1.6	0.010865434
HALLMARK_CHOLESTEROL_HOMEOSTASIS	1.6	0.01064314
HALLMARK_ALLOGRAFT_REJECTION	1.4	0.06476901
HALLMARK_PEROXISOME	1.4	0.08273839
HALLMARK_GLYCOLYSIS	1.3	0.08865474
HALLMARK_COMPLEMENT	1.3	0.09164486
HALLMARK_COAGULATION	1.3	0.10120447
HALLMARK_APOPTOSIS	1.3	0.09475186
HALLMARK_IL2_STAT5_SIGNALING	1.2	0.15677549
HALLMARK_ESTROGEN_RESPONSE_EARLY	1.2	0.17921966
HALLMARK_NOTCH_SIGNALING	1.2	0.2382772
HALLMARK_KRAS_SIGNALING_DN	1.2	0.2343269

f. LUAD High CD274 Enriched Gene Sets

LUAD High CD274 Enriched Gene Sets (25% FDR)	NES	FDR q-val
HALLMARK_INTERFERON_GAMMA_RESPONSE	3.9	0
HALLMARK_ALLOGRAFT_REJECTION	3.8	0
HALLMARK_INFLAMMATORY_RESPONSE	3.5	0
HALLMARK_TNFA_SIGNALING_VIA_NFKB	3.5	0
HALLMARK_INTERFERON_ALPHA_RESPONSE	3.4	0
HALLMARK_IL6_JAK_STAT3_SIGNALING	3.2	0
HALLMARK_COMPLEMENT	2.9	0
HALLMARK_IL2_STAT5_SIGNALING	2.7	0
HALLMARK_KRAS_SIGNALING_UP	2.7	0
HALLMARK_APOPTOSIS	2.5	0
HALLMARK_APICAL_JUNCTION	2.4	0
HALLMARK_TGF_BETA_SIGNALING	2.2	0
HALLMARK_EPITHELIAL_MESENCHYMAL_TRANSITION	2.1	0
HALLMARK_HYPOXIA	1.9	1.02E-04
HALLMARK_COAGULATION	1.9	3.24E-04
HALLMARK_UV_RESPONSE_DN	1.8	5.87E-04
HALLMARK_APICAL_SURFACE	1.7	0.003995385
HALLMARK_ANGIOGENESIS	1.6	0.006372829
HALLMARK_P53_PATHWAY	1.5	0.012783593
HALLMARK_PI3K_AKT_MTOR_SIGNALING	1.5	0.023198167
HALLMARK_MYOGENESIS	1.4	0.030198675
HALLMARK_HEME_METABOLISM	1.4	0.047247607
HALLMARK_HEDGEHOG_SIGNALING	1.4	0.050490916
HALLMARK_MITOTIC_SPINDLE	1.3	0.08157678
HALLMARK_CHOLESTEROL_HOMEOSTASIS	1.2	0.15549049
HALLMARK_UV_RESPONSE_UP	1.2	0.1805793

g. LUAD Low PRSS8 Enriched Gene Sets

LUAD Low PRSS8 Enriched Gene Sets (25% FDR)	NES	FDR q-val
HALLMARK_E2F_TARGETS	-3.1	0
HALLMARK_G2M_CHECKPOINT	-3.0	0
HALLMARK_MYC_TARGETS_V1	-2.5	0
HALLMARK_SPERMATOGENESIS	-2.1	0
HALLMARK_MITOTIC_SPINDLE	-2.0	0
HALLMARK_EPITHELIAL_MESENCHYMAL_TRANSITION	-2.0	1.16E-04
HALLMARK_MYC_TARGETS_V2	-1.9	5.12E-04
HALLMARK_MTORC1_SIGNALING	-1.8	0.001880215
HALLMARK_UV_RESPONSE_DN	-1.5	0.034481023
HALLMARK_UNFOLDED_PROTEIN_RESPONSE	-1.3	0.12530452
HALLMARK_OXIDATIVE_PHOSPHORYLATION	-1.3	0.16235016

h. LUAD Low CD274 Enriched Gene Sets

LUAD Low CD274 Enriched Gene Sets (25% FDR)	NES	FDR q-val
HALLMARK_OXIDATIVE_PHOSPHORYLATION	-2.6	0
HALLMARK_MYC_TARGETS_V1	-2.4	0
HALLMARK_E2F_TARGETS	-2.0	0
HALLMARK_MYC_TARGETS_V2	-1.9	5.70E-04
HALLMARK_UNFOLDED_PROTEIN_RESPONSE	-1.7	0.004312027
HALLMARK_FATTY_ACID_METABOLISM	-1.6	0.010758515
HALLMARK_G2M_CHECKPOINT	-1.6	0.012803096
HALLMARK_SPERMATOGENESIS	-1.6	0.011908125
HALLMARK_GLYCOLYSIS	-1.5	0.02734927
HALLMARK_DNA_REPAIR	-1.4	0.033594772
HALLMARK_ADIPOGENESIS	-1.4	0.042960566
HALLMARK_MTORC1_SIGNALING	-1.3	0.079123646
HALLMARK_XENOBIOTIC_METABOLISM	-1.3	0.09165737
HALLMARK_PANCREAS_BETA_CELLS	-1.3	0.12316957
HALLMARK_PEROXISOME	-1.2	0.16723569
HALLMARK_ESTROGEN_RESPONSE_LATE	-1.2	0.19401155

Supplementary Figure 4

Pathway Comparison for PRSS8 and PD-L1 in LUSC and LUAD

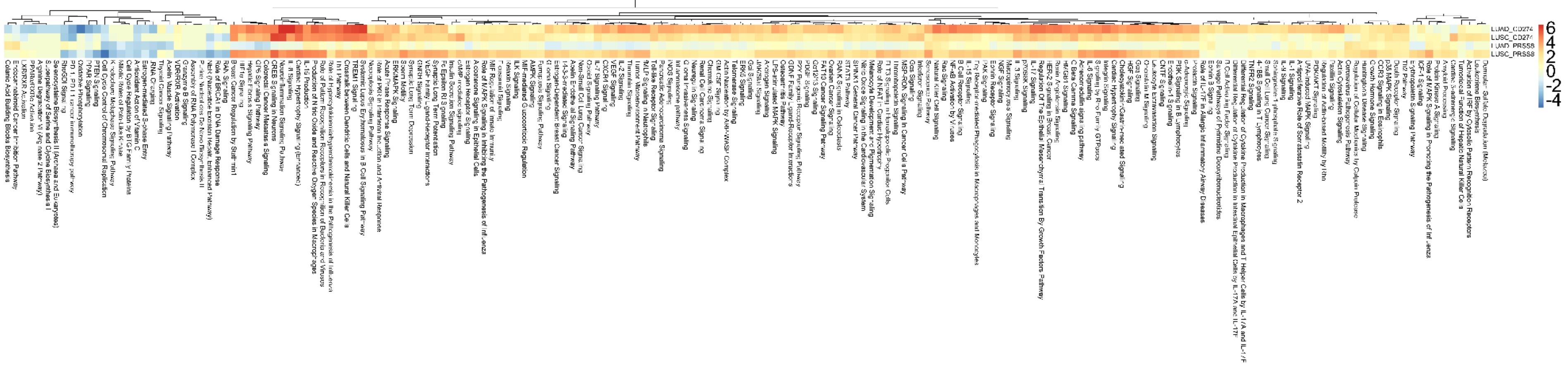

Supplementary Figure 5 (a-g, uncropped Western blot images)

All images are overlays of ink-stained membrane (with molecular weight markers) and the corresponding chemiluminescent blot.

Supplementary Figure 5a: Figure 1_uncropped images

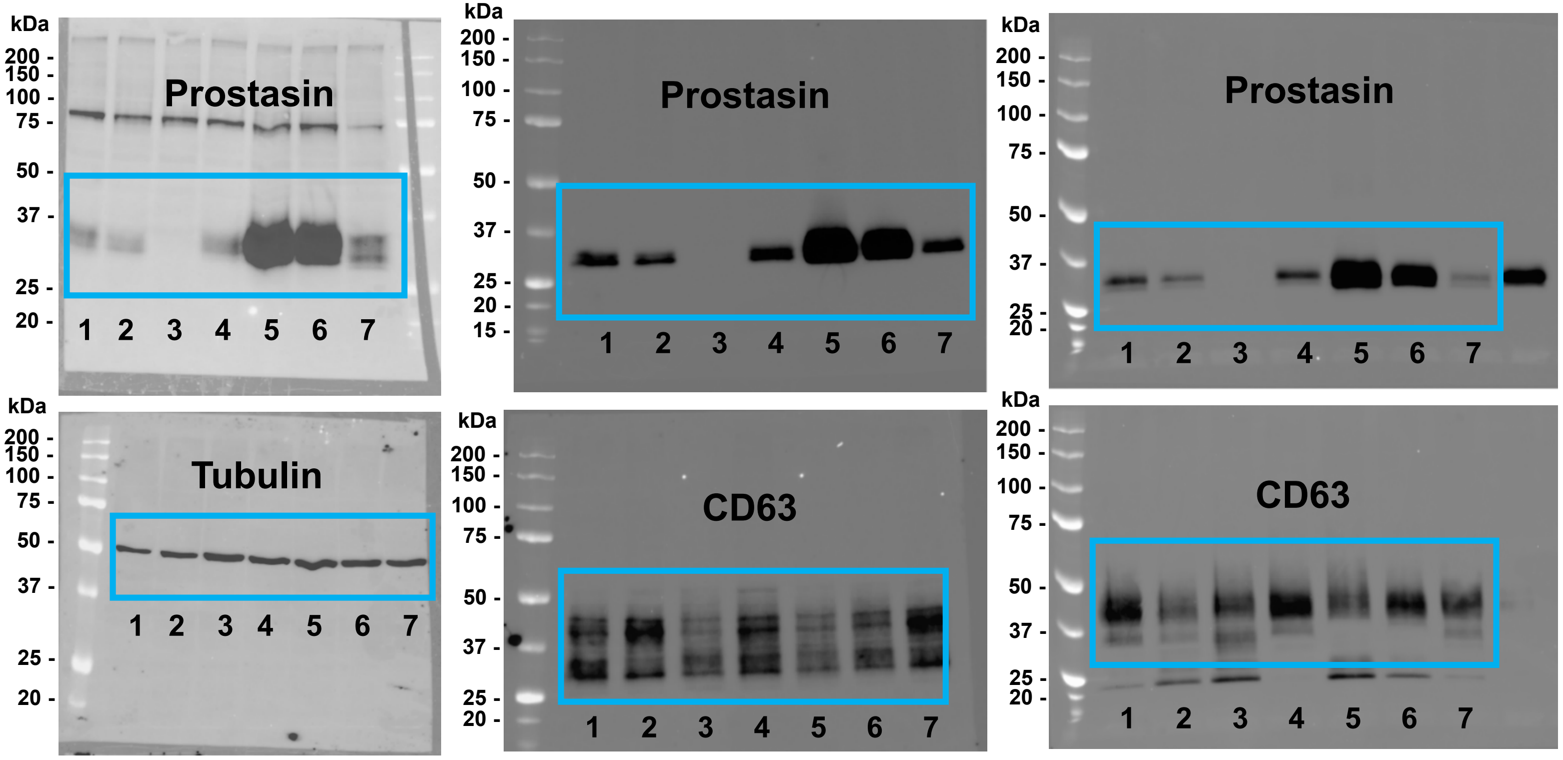

Supplementary Figure 5b: Figure 2_uncropped images

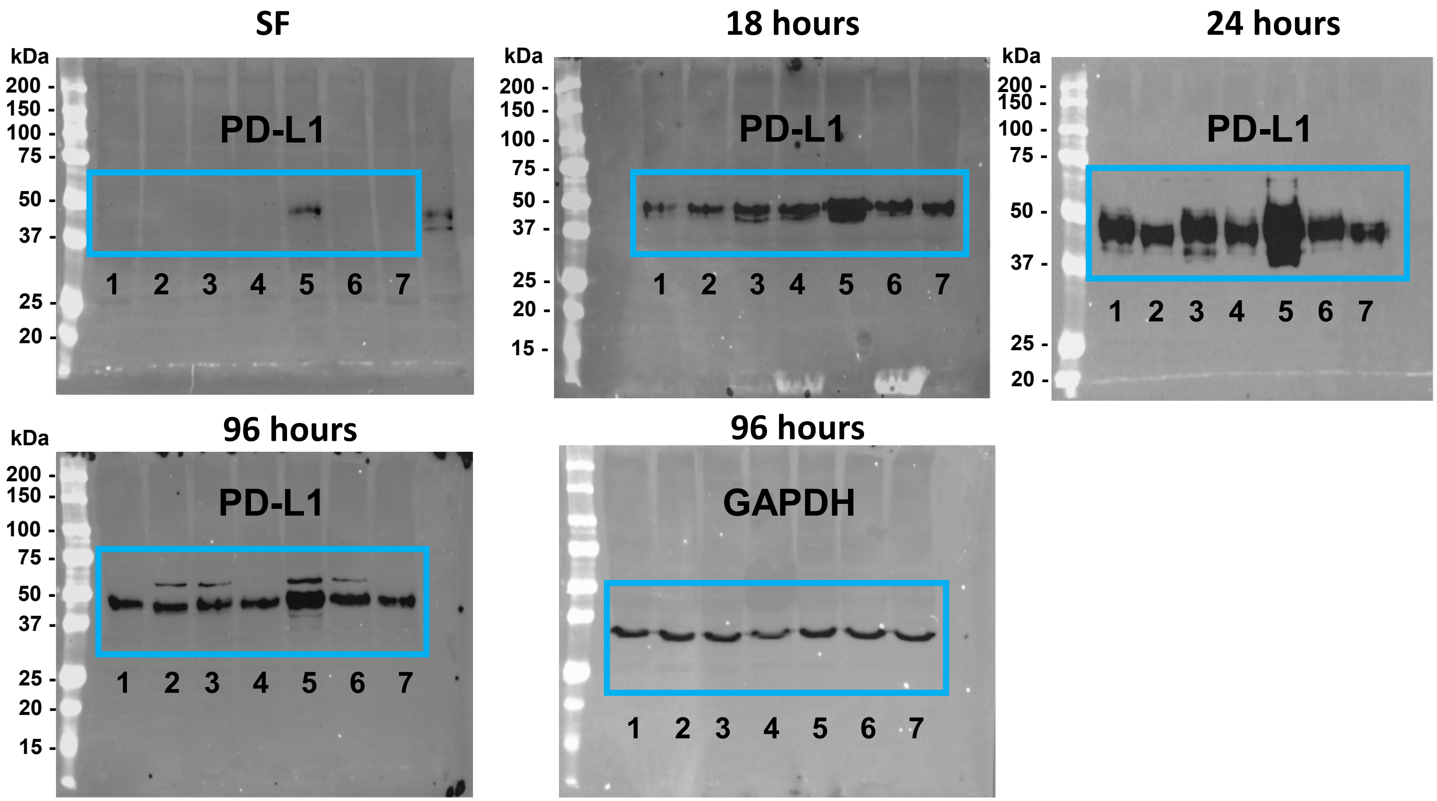

Supplementary Figure 5c: Figure 4_uncropped images

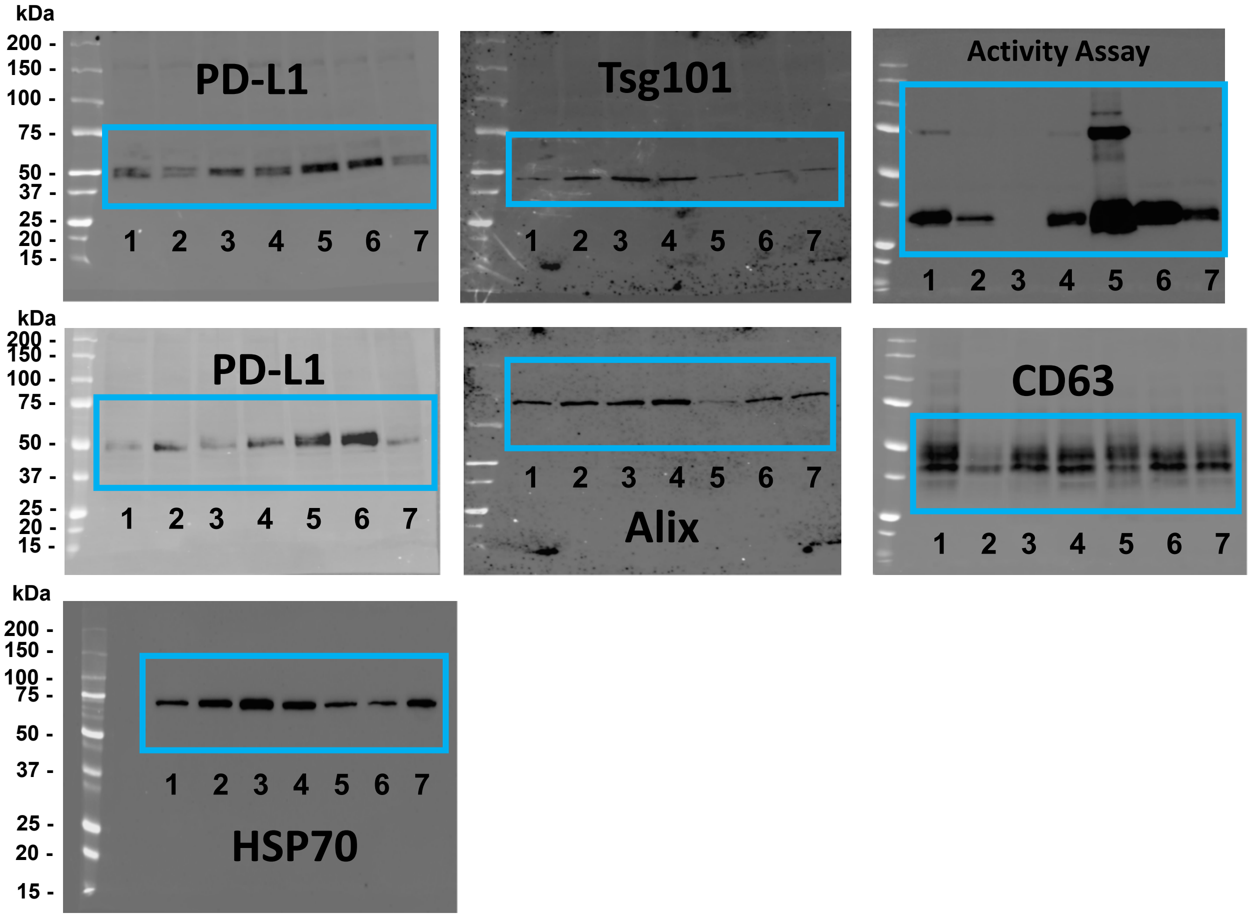

Supplementary Figure 5d: Figure 5_uncropped images

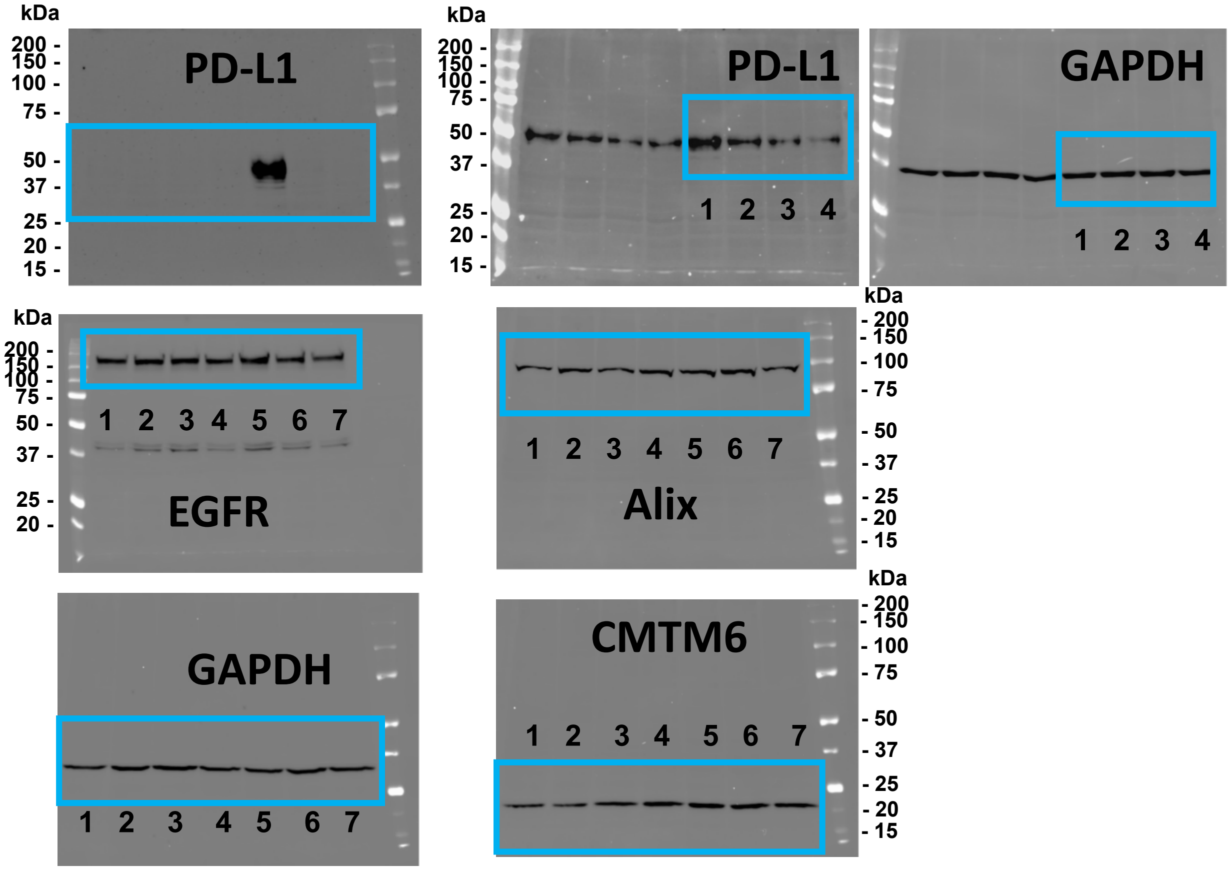

Supplementary Figure 5e: Figure 6_uncropped images

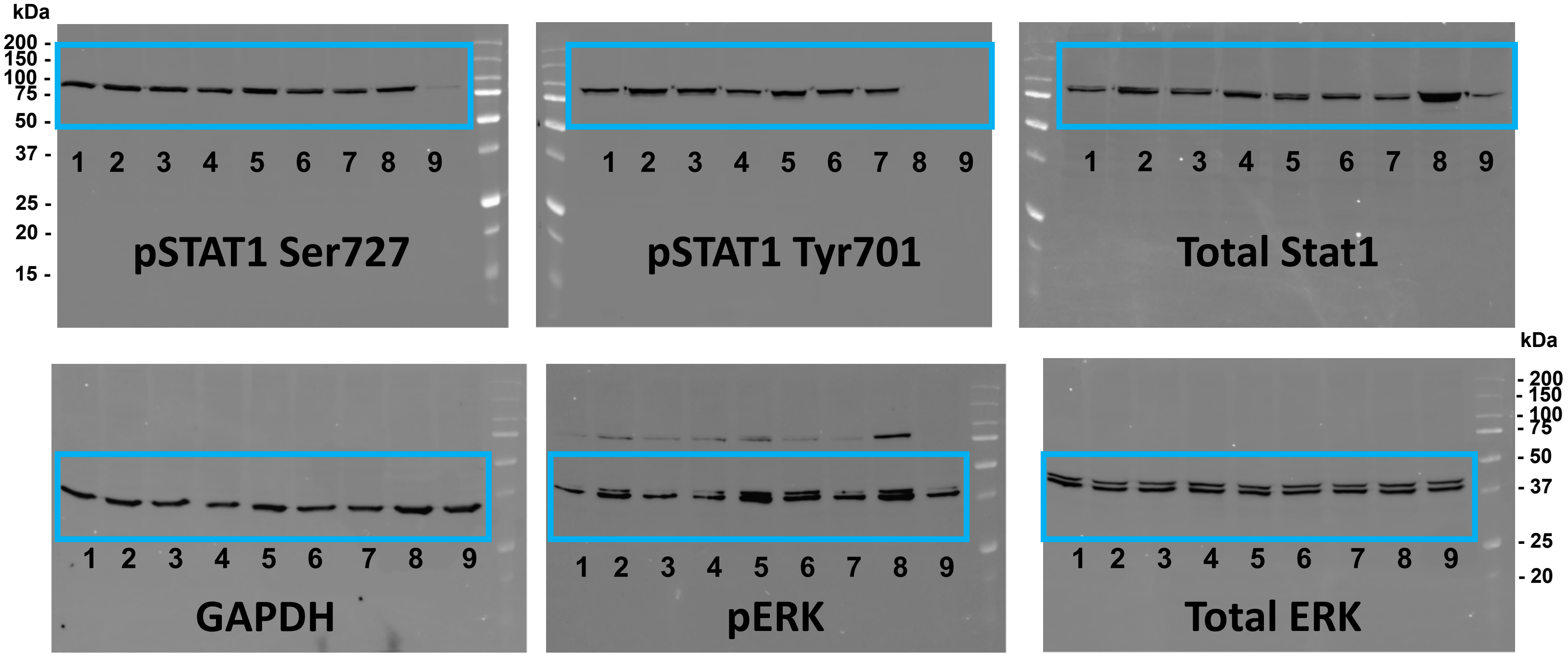

Supplementary Figure 5f: Figure 7_uncropped images

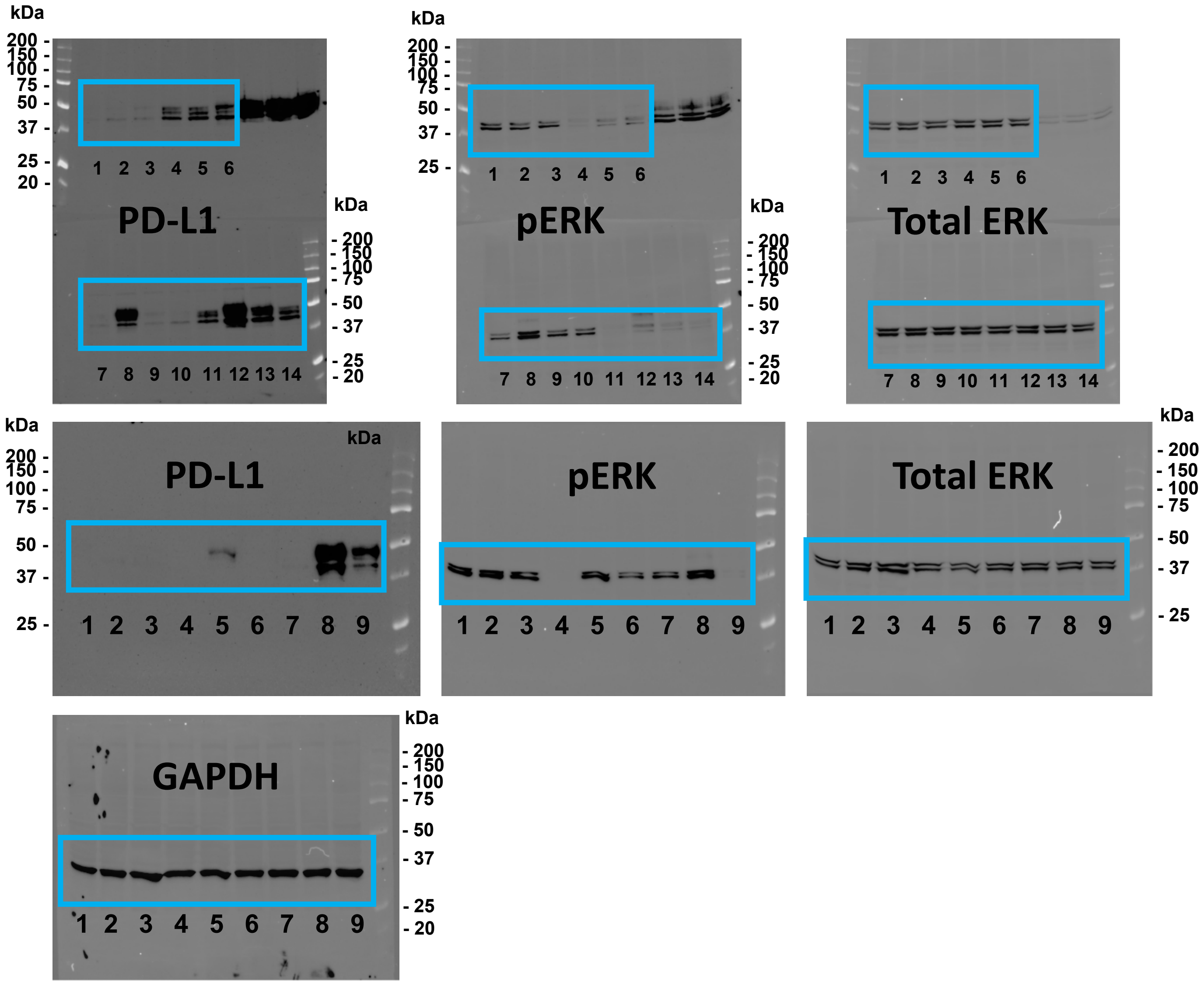

Supplementary Figure 5g: Figure 8_uncropped images

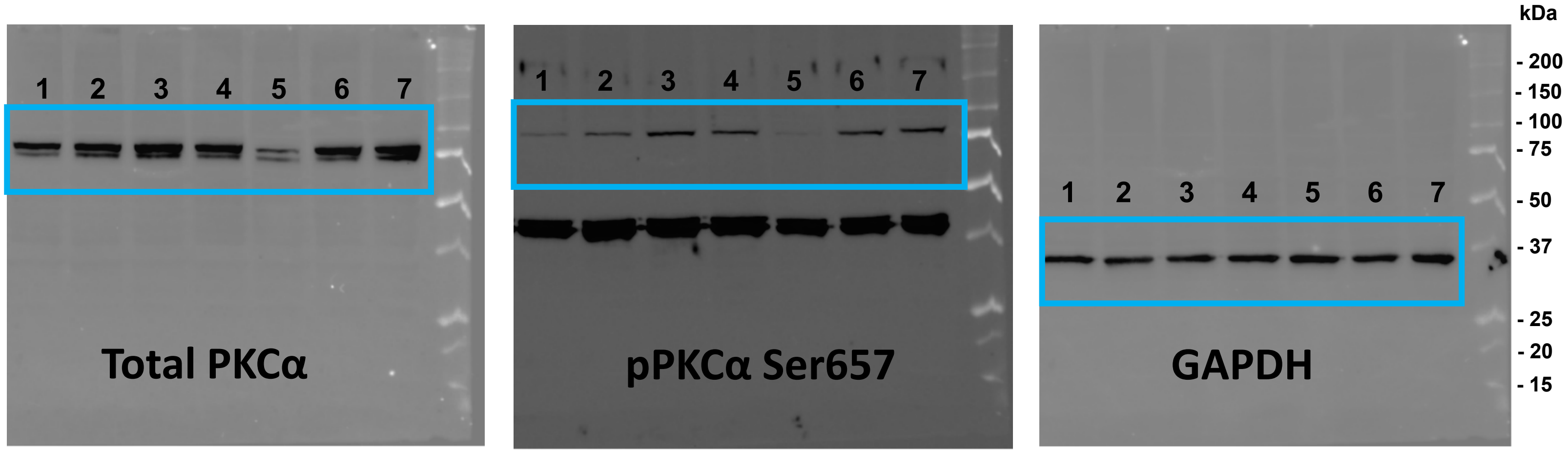

Supplement: Supplementary Figures S1-S5 [file BSR-2021-1370_supp.pdf]
